# *miR-655* Is an EMT-Suppressive MicroRNA Targeting *ZEB1* and *TGFBR2*

**DOI:** 10.1371/journal.pone.0062757

**Published:** 2013-05-14

**Authors:** Yosuke Harazono, Tomoki Muramatsu, Hironori Endo, Narikazu Uzawa, Tatsuyuki Kawano, Kiyoshi Harada, Johji Inazawa, Ken-ichi Kozaki

**Affiliations:** 1 Department of Molecular Cytogenetics, Tokyo Medical and Dental University, Tokyo, Japan; 2 Department of Therapeutic Genomics, Medical Research Institute and School of Biomedical Science, Tokyo Medical and Dental University, Tokyo, Japan; 3 Department of Genome Medicine, Hard Tissue Genome Research Center, Tokyo Medical and Dental University, Tokyo, Japan; 4 Global Center of Excellence (GCOE) Program for International Research Center for Molecular Science in Tooth and Bone Diseases, Tokyo Medical and Dental University, Tokyo, Japan; 5 Department of Maxillofacial Surgery, Tokyo Medical and Dental University, Tokyo, Japan; 6 Department of Esophagogastric Surgery, Tokyo Medical and Dental University, Tokyo, Japan; The Ohio State University, United States of America

## Abstract

Recently, the epithelial-to-mesenchymal transition (EMT) has been demonstrated to contribute to normal and disease processes including cancer progression. To explore EMT-suppressive microRNAs (miRNAs), we established a cell-based reporter system using a stable clone derived from a pancreatic cancer cell line, Panc1, transfected with a reporter construct containing a promoter sequence of *CDH1/E-cadherin* in the 5′ upstream region of the *ZsGreen1* reporter gene. Then, we performed function-based screening with 470 synthetic double-stranded RNAs (dsRNAs) mimicking human mature miRNAs using the system and identified *miR-655* as a novel EMT-suppressive miRNA. Overexpression of *miR-655* not only induced the upregulation of E-cadherin and downregulation of typical EMT-inducers but also suppressed migration and invasion of mesenchymal-like cancer cells accompanied by a morphological shift toward the epithelial phenotype. In addition, we found a significant correlation between *miR-655* expression and a better prognosis in esophageal squamous cell carcinoma (ESCC). Moreover, *ZEB1* and *TGFBR2*, which are essential components of the TGF-b signaling pathway, were identified as direct targets of *miR-655*, suggesting that the activation of the TGF-b-ZEB1-E-cadherin axis by aberrant downregulation of *miR-655* may accelerate cancer progression.

## Introduction

The epithelial-to-mesenchymal transition (EMT) is an essential biological process with remarkable morphological changes between the epithelial and mesenchymal states [1], and plays key roles in embryonic development, cancer and other diseases [2]–[5]. During the acquisition of EMT characteristics, cancer cells lose the expression of genes that promote cell-cell contact, such as *E-cadherin* and the *miR-200* family, and gain the expression of mesenchymal markers, such as *vimentin*, *fibronectin*, and *N-cadherin*, leading to enhanced cancer cell migration and invasion [6]–[8] and to confer drug resistance characteristics on cancer cells [9]. Therefore, the development of EMT inhibitors may provide novel strategies for the prevention, diagnosis and treatment of cancers.

MicroRNAs (miRNAs) are endogenous small non-protein-coding RNAs of 19–22 nucleotides. These single-stranded RNAs are considered to play crucial roles in many normal cellular processes [10], [11], [12], and the multistep processes of carcinogenesis and cancer progression [13]–[15], depending on their specific gene targets. Furthermore, The many achievements in the field of the discovery of tumor-suppressive miRNAs (TS-miRNAs) and *in vitro*/*in vivo* delivery technology may offer the possibility of new therapeutic approaches for cancer. Since one miRNA can target an unpredictable number of messenger RNAs (mRNAs) of protein-coding genes on a genome-wide scale, the clinical applications of miRNAs for cancer therapies are considered better than those of short interfering RNAs (siRNAs). In addition, among miRNA-based approaches by *in vivo* delivery including the use of DNA plasmids or viral vectors, miRNA replacement therapy using double-stranded RNAs (dsRNAs) mimicking TS-miRNAs may be one of the most promising, offering hope for new cancer therapies [15], [16].

Recently, the *miR-200* family (*miR-141*, *-200a*, *-200b, -200c*, and *-429*) and *miR-205* have been demonstrated as EMT-suppressive miRNAs directly targeting *ZEB1* and *ZEB2* [17]. The *miR-200*-ZEB1-E-cadherin axis has been clarified to be a crucial pathway downstream of TGF-b in EMT while reciprocal repression between *ZEB1* and the *miR-200* family has recently been reported to promote EMT and invasion in cancer cells [18]–[22]. Actually, EMT-induced cancer cells were also reported to be more efficient at forming cancer stem cells with invasive and tumorigenic phenotypes [23]. Therefore, EMT-suppressive miRNAs in cancers have been considered to be important diagnostic markers and new therapeutic agents for human malignancies.

Herein, we show the identification of a novel EMT-suppressive miRNA by function-based screening using 470 synthetic miRNAs and the detailed characterization of the miRNA and its direct targets. The function-based screening makes it possible to analyze the biological effects of a large number of dsRNAs on cancer cells directly. In addition, this approach has already proved successful in the exploration of dsRNAs having oncogenic or tumor-suppressive effects on cancer cells [24]–[27]. In the present study, to detect the promoter activity of *CDH1/E-cadherin* by measuring the fluorescence intensity of ZsGreen1 protein in our function-based screening, we established a unique cell-based reporter system using a pancreatic cancer cell line, Panc1, having phenotypic plasticity at EMT/mesenchymal-to-epithelial transition (MET). The present study is the first to show clearly that *miR-655* targets *ZEB1* and *TGFBR2* inducing inactivation of the TGF-b signaling pathway, involving the *miR-200*-ZEB1-E-cadherin axis, strongly suggesting a potential role for *miR-655* as a prognostic marker and therapeutic agent in human cancers.

## Materials and Methods

### Cell Lines and Primary Tumor Samples

The culture conditions for the pancreatic cancer [28], esophageal squamous-cell carcinoma (ESCC) [29] and oral squamous cell carcinoma (OSCC) [30], [31] cell lines were reported previously. These cell lines were authenticated in previous studies with array-based comparative genomic hybridization (aCGH) analyses [28], [29]. A breast cancer cell line, MDA-MB-231, was purchased from the American Type Culture Collection (Manassas, VA, USA) and maintained in the medium recommended by the manufacturers. Primary ESCCs and OSCCs were obtained with the written consent of each patient after approval by a local ethics committee of Medical Research Institute and Faculty of Medicine, Tokyo Medical and Dental University (Approval ID: 2010-5-2).

### Transfection with Synthetic miRNAs and Small Interfering RNAs (siRNAs)

10 nM of dsRNA mimicking human mature miRNA or control non-specific miRNA (Ambion, Austin, TX; Thermo Scientific Dharmacon, Lafayette, CO) was transfected individually into cells using Lipofectamine RNAiMAX (Invitrogen, Carlsbad, CA). The function-based screening was performed using Pre-miR™ miRNA Precursor Library-Human V3 (Ambion) in duplicate [26], [27]. The numbers of viable cells were assessed by the colorimetric water-soluble tetrazolium salt (WST-8) assay (Cell counting kit-8; Dojindo Laboratories, Kumamoto, Japan). The reporter construct was generated using the pZsGreen1-1 Vector (Clontech Laboratories, Palo Alto, CA). The fluorescence intensity of the ZsGreen1 protein was measured by ARVO mx (Perkin Elmer, Waltham, MA).

### Transwell Migration and Invasion Assay

Transwell migration and invasion assays were carried out in 24-well modified chambers precoated with (invasion) or without (migration) Matrigel (BD BioCoat, BD Biosciences, Franklin Lakes, NJ) as described elsewhere [32]. Cells in serum-free medium were transferred into the upper chambers. After incubation, the cells that migrated into the lower chambers with 10% FBS as the chemoattractant were fixed and stained with the Diff-Quik stain (Sysmex, Kobe, Japan), and counted in 5 random fields. Each assay was performed in triplicate.

### Real-time Reverse Transcription-PCR and miRNA Target Predictions

Real-time reverse transcription-PCR (RT-PCR) was performed as described elsewhere [33]. Predicted targets for miRNAs and their target sites were analyzed using microRNA.org. All samples were analyzed in a duplicated manner.

### Western Blot Analysis and Luciferase Activity Assay

An anti-CDH1 (#610181) antibody (BD Biosciences), an anti-TGFBR2 (SC-220), an anti-TGFBR1 (SC-398) antibody (Santa Cruz Biotechnology, Santa Cruz, CA), an anti-ZEB1 (#3396S), an anti-Snail1 (#3879S), an anti-Phospho-Smad2/3 (Ser465/467 and Ser423/425, respectively) (#3101 and #9520, respectively) antibodies (Cell Signaling Technology, Beverly, MA), an anti-Smad2/3 (ab40855 and ab28379, respectively) antibodies (Abcam, Cambridge, MA) were used in Western blotting. Immunohistochemistry was performed as described elsewhere [32]. Luciferase constructs were made by ligating oligonucleotides containing the wild type or mutated sequence of 3′-UTR target sites downstream of the luciferase gene in the pMIR-REPORT luciferase vector (Ambion). Luciferase activity was measured as described elsewhere [33].

### Statistical Analysis

The association between clinicopathological characteristics and status of *miR-655* expression in ESCC patients was evaluated with χ^2^ or Fisher’s exact test (Table S2). A *p*-value less than 0.05 was defined as being statistically significant. In Kaplan-Meier curves, differences between subgroups were tested with the log-rank test. Differences between subgroups were tested with the Mann-Whitney *U* test.

## Results

### Establishment of a Cell-based Reporter System for Investigating *CDH1/E-cadherin*-promoter Activity in Panc1 Cells

**Figure 1:**
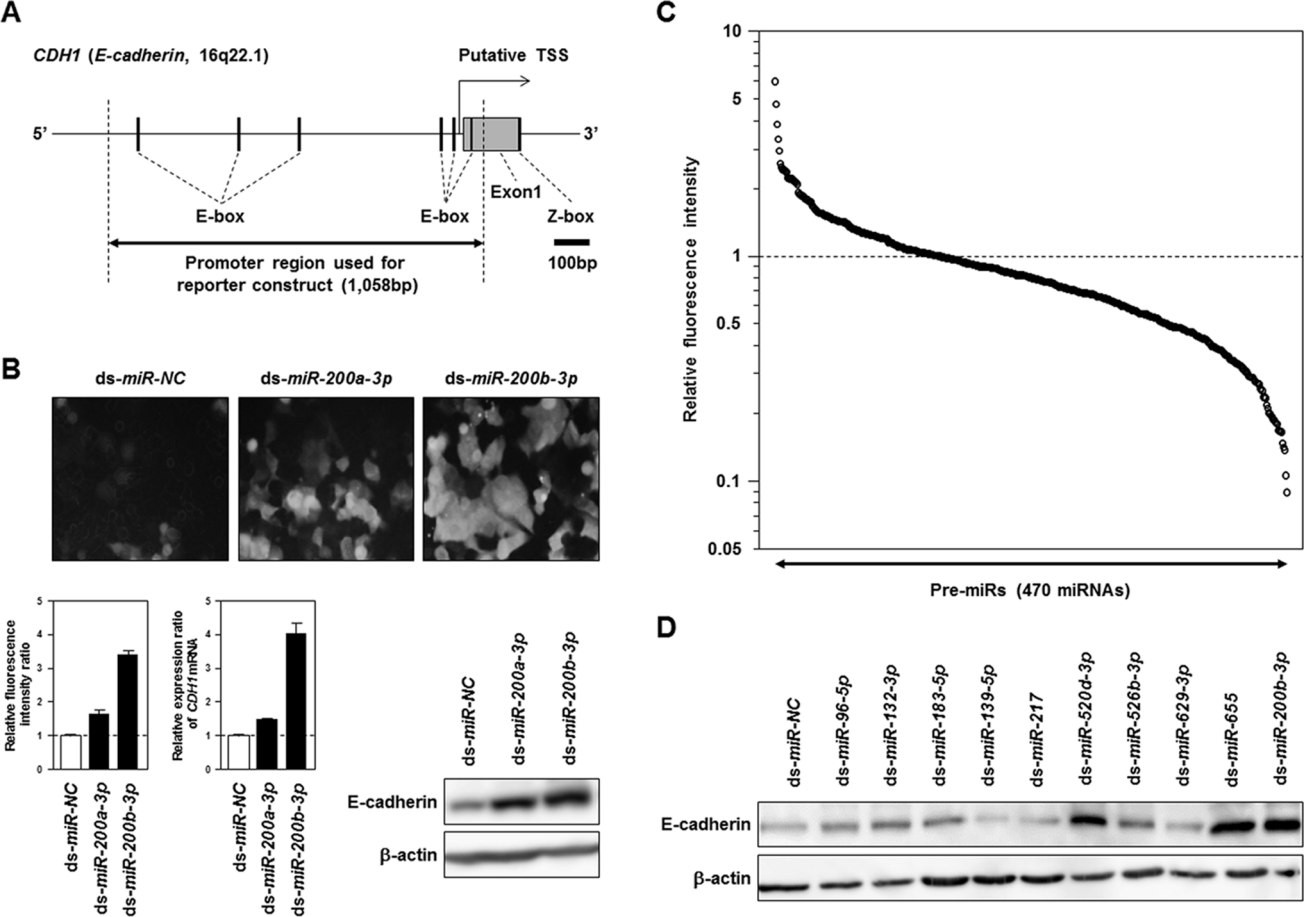
Function-based screening of EMT-suppressive miRNAs using reporter system for investigating *CDH1/E-cadherin*-promoter activity in Panc1 cells. ***A***, Map of the promoter region of the *CDH1/E-cadherin* gene. To construct a reporter plasmid, 1,058 bp promoter sequences indicated by the closed arrow in this map was introduced into a promoterless pZsGreen1-1 vector with the *ZsGreen1* gene as a reporter gene. A cell-based reporter system was established by isolation of a stable clone with the limiting dilution method after transfection of the construct into Panc1 cells. ***B***, Confirmation of the expression of the ZsGreen1 protein in the cell-based reporter system following transfection of *miR-200a* or *-200b*. A stable cell clone with the reporter plasmid was evaluated 96 hours after transient transfection of 10 nM of dsRNA mimicking *miR-200a* or *-200b,* or control non-specific miRNA (ds-*miR-200a*, ds-*miR-200b* or ds-*NC*) (Ambion). ***Upper*** **,** Detection of ZsGreen1 in these transfectants using fluorescence micrographs. ***Lower*** **,** Quantification of fluorescence intensity in these transfectants (***Left***). Results of the TaqMan real-time RT-PCR analysis (***Middle***) and Western blot analysis (***Right***) for expression of the *CDH1/E-cadherin* transcript and protein, respectively, in these transfectants. ***C***, Results of the function-based screening of EMT-suppressive miRNAs in a cell-based reporter system using Pre-miR™ miRNA Precursor Library-Human V3 (Ambion) containing 470 dsRNAs mimicking human mature miRNAs. The fluorescence intensity of ZsGreen1 was evaluated by fluorescence microplate reader in duplicate. The relative fluorescence intensity in each transfectant was calculated by normalization of each result to the fluorescence intensity in control cells transfected with non-specific miRNA (see Table 1 and Table S2). The lower closed arrow indicates the 470 miRNAs examined. ***D***, Western blot analysis of E-cadherin protein levels in parental Panc1 cells 96 hours after transient transfection with 10 nM of ds-*NC* or 10 nM of ds-miRNAs mimicking *miR-96-5p, -132-3p, -183-5p, -139-5p, -217, -520d-3p, -526b-3p, -629-3p, -655* and *-200b-3p*. Because *miR-200b* has already been confirmed to induce expression of the *CDH1/E-cadherin* transcript and protein in this study (Fig. 1B) and multiple previous studies, ds-*miR-200b* was used as a positive control in this analysis.

To perform function-based screening of EMT-suppressive miRNAs, we established a cell-based reporter system. The promoter region of the *CDH1/E-cadherin* gene located in the 5′ untranslated region (5′UTR) and Exon1 (from nt −1001 to +57 relative to the transcription start site, TSS, at the 5′end of the gene) was prepared by genomic PCR using specific primers (Table S1), and was inserted into the pZsGreen1-1 vector at the multiple cloning site upstream of the promoterless *ZsGreen1* gene (Fig. 1A) [34]. We first transfected the above-mentioned construct into Panc1 cells, the phenotypic plasticity of which had already been confirmed in previous studies [35], [36], and then carried out cloning of stable transfectants by limiting dilution. Furthermore, among many clones, the most-reactive single-cell clone, PEcadZsG-Panc1, was selected by measuring the fluorescence intensity of the ZsGreen1 protein induced by transient transfection of *miR-200a* or *-200b*, already known as EMT-regulating miRNAs. In this clone 96 hours after transfection with 10 nM of dsRNA mimicking *miR-200a* or *-200b*, the fluorescence intensity of ZsGreen1 was remarkably increased as compared with that in the control counterparts (Fig. 1B). RT-PCR and Western blot analyses also showed a consistent correlation between the fluorescence intensity of ZsGreen1 and expression levels of *CDH1/E-cadherin* mRNA and protein in these transfectants. Consequently, we judged that ZsGreen1 expression was tightly regulated under the *CDH1/E-cadherin*-promoter in our cell-based reporter system for function-based screening of EMT-suppressive miRNAs.

### Function-based Screening of EMT-suppressive miRNAs with our cell-based Reporter System

To identify EMT-suppressive miRNAs, we performed function-based screening, in which the fluorescence intensity of ZsGreen1 was made an index, using our cell-based reporter system and 470 dsRNAs at 10 nM. Figure 1C and Table S2 demonstrate results of this screening in a Panc1 stable clone, PEcadZsG-Panc1, 96 hours after transient transfection with each dsRNA. In Table 1, 17 miRNAs, the relative fluorescence intensity of which remarkably increased in our screening (>2.2-fold change of mean fluorescence intensity compared with the control counterpart), were enrolled as candidate EMT-suppressive miRNAs. Among these candidates, we excluded well-known EMT-suppressive miRNAs, such as *miR-200a* and *-200c* [8], [21], [37], [38], and selected 10 miRNAs (*miR-96-5p, -132-3p, -183-5p, -139-5p, -217, -520d-3p, -526b-3p, -629-3p, -655*, and *-200b-3p*) showing a consistent positive correlation between two sets of fluorescence data taken from the fluorescence microplate reader (Table S2) and fluorescence microscope (Fig. S1). In a Western blot analysis in the parental Panc1 cells 96 hours after transient transfection with these miRNAs, *miR-520d-3p* and *miR-655*, as well as *miR-200b-3p*, were confirmed to upregulate expression of the E-cadherin protein markedly, whereas only slight effects of other miRNAs were observed (Fig. 1D). Moreover, the relative fluorescence intensity of *miR-655* was clearly higher than that of *miR-520d-3p* (Table 1), suggesting *miR-655* to be a prime candidate for EMT-suppressive miRNA.

**Table 1:** Summary of 17 miRNA genes selected as candidates for EMT-suppressive miRNAs in functional-based screening using a stable Panc1 clone transfected with a reporter construct containing a promoter sequence of *CDH1/E-cadherin* in the 5′ upstream region of the *ZsGreen1* reporter gene and Pre-miR™ miRNA Precursor Library - Human V3 (Ambion).

			Ratio of fluorescence intensity of ZsGreen1 (RFI)*		Ratio of growth level (RG)**		Relative fluorescence intensity (RFI/RG)	
	Pre-miR™ miRNA Precursor	Mature Sequence	Mean	SD	Mean	SD	Mean	SD
1	*hsa-miR-200c-3p*	UAAUACUGCCGGGUAAUGAUGG	5.22	0.05	0.86	0.08	5.99	0.15
2	*hsa-miR-200b-3p*	UAAUACUGCCUGGUAAUGAUGAC	4.91	0.09	1.06	0.14	4.74	0.47
3	*hsa-miR-655*	AUAAUACAUGGUUAACCUCUUU	4.09	0.00	1.06	0.06	3.87	0.07
4	*hsa-miR-200a-3p*	UAACACUGUCUGGUAACGAUGU	4.22	0.03	1.28	0.04	3.30	0.16
5	*hsa-miR-132-3p*	UAACAGUCUACAGCCAUGGUCG	2.53	0.02	0.83	0.08	2.94	0.01
6	*hsa-miR-526b-3p*	AAAGUGCUUCCUUUUAGAGGC	3.27	0.03	1.28	0.02	2.58	0.06
7	*hsa-miR-302c-3p*	UAAGUGCUUCCAUGUUUCAGUGG	2.37	0.15	1.36	0.04	2.49	0.19
8	*hsa-miR-373-3p*	GAAGUGCUUCGAUUUUGGGGUGU	2.93	0.11	1.20	0.06	2.43	0.21
9	*hsa-miR-217*	UACUGCAUCAGGAACUGAUUGGAU	2.66	0.06	1.11	0.04	2.42	0.20
10	*hsa-miR-629-3p*	GUUCUCCCAACGUAAGCCCAGC	3.43	0.16	0.96	0.05	2.40	0.96
11	*hsa-miR-302d-3p*	UAAGUGCUUCCAUGUUUGAGUGU	3.08	0.04	1.29	0.16	2.37	0.11
12	*hsa-miR-361-5p*	UUAUCAGAAUCUCCAGGGGUAC	2.35	0.15	1.00	0.16	2.36	0.13
13	*hsa-miR-96-5p*	UUUGGCACUAGCACAUUUUUGC	1.63	0.18	1.43	0.12	2.23	0.05
14	*hsa-miR-139-5p*	UCUACAGUGCACGUGUCU	2.30	0.06	1.06	0.03	2.22	0.16
15	*hsa-miR-183-5p*	UAUGGCACUGGUAGAAUUCACUG	2.76	0.01	1.24	0.00	2.21	0.01
16	*hsa-miR-520d-3p*	AAAGUGCUUCUCUUUGGUGGGUU	1.57	0.69	1.18	0.09	2.20	0.30
17	*hsa-miR-181d*	AACAUUCAUUGUUGUCGGUGGGUU	2.27	0.01	1.29	0.06	2.20	0.00

* The ratio of fluorescence intensity of ZsGreen1 (RFI) in cells 4 days after transfection with each dsRNA was normalized to that in control transfectants (Pre-miRTM Negative Control #1, Ambion).

** The ratio of growth level (RG) of viable cells assessed by WST8 assay 4 days after transfection with dsRNAs. WST-8 assay was employed to normalize the number of viable cells relative to the control transfectants.

### EMT-suppressive Effects of *miR-655* on Mesenchymal-like Cancer Cells having Phenotypic Plasticity at EMT/MET

**Figure 2:**
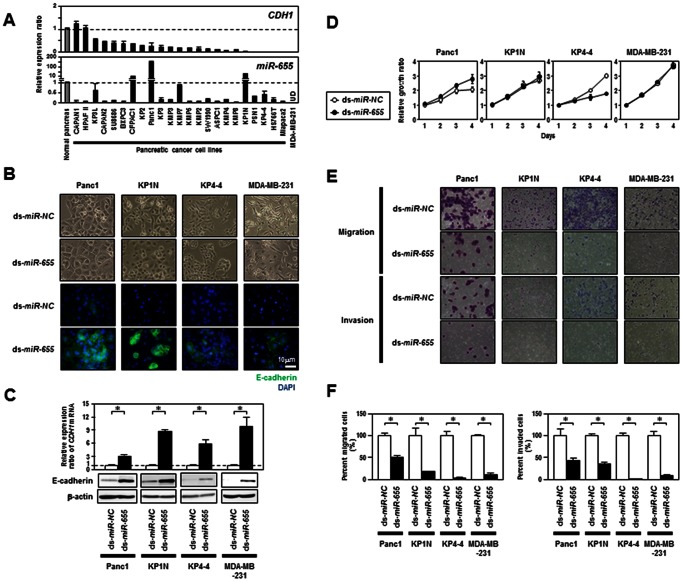
EMT-suppressive effects of *miR-655* on mesenchymal-like cancer cells having phenotypic plasticity at EMT/MET. ***A***, TaqMan real-time RT-PCR analysis of *CDH1/E-cadherin* and *miR-655* in a panel of 23 pancreatic cancer cell lines and a breast cancer cell line, MDA-MB-231. Relative expression levels of transcripts of *CDH1/E-cadherin* and *miR-655* were quantified in comparison to *GAPDH* and *RNU6B*, respectively, to normalize the initial input of total RNA. Bar graphs show the ratio of the expression level in these cell lines to that in normal pancreas (Ambion). ***B***, Representative results of phase contrast images (***Upper***) and *CDH1/E-cadherin* protein expression level detected by immunofluorescence staining (***Lower***) in Panc1, KP1N, KP4-4 and MDA-MB-231 cells 96 hours after transfection with 10 nM of ds-*NC* or dsRNA mimicking *miR-655* (ds-*miR-655*) (Ambion). ***C***, TaqMan real-time RT-PCR analysis (***Upper***) and Western blot (***Lower***) analysis of mRNA and protein levels of *CDH1/E-cadherin*, respectively, in Panc1, KP1N, KP4-4 and MDA-MB-231 cells 96 hours after transfection of 10 nM of ds-*NC* or ds-*miR-655*. Asterisks (*), statistical analysis with the Mann-Whitney *U* test. ***D***, Growth curves in Panc1, KP1N, KP4-4 and MDA-MB-231 cells after transfection of 10 nM of ds-*NC* or ds-*miR-655*. Each data point represents the mean of duplicate determinations (bars, SD) in these experiments. Asterisks (*), statistical analysis with the Mann-Whitney *U* test. ***E***, Representative phase micrographs of Panc1, KP1N, KP4-4 and MDA-MB-231 cells transiently transfected with 10 nM of ds-*NC* or ds-*miR-655* in cell migration and invasion assays *in vitro* using uncoated and Matrigel-coated transwell-chamber culture systems (Becton Dickinson), respectively. At 48 hours after transfection of dsRNA, cells were transferred into the upper chamber of the transwell (4×10^4^ cells per well). The migrating or invading cells on the lower surface of filters were fixed and stained with the Diff-Quik stain 48 hours after cell transfer. ***F***, Quantification of the cell migration (***Left***) and invasion (***Right***) shown in Figure 3B. Bar graphs show the percentage (%) of *miR-655*-transfectants migrating (***Left***) or invading (***Right***) through uncoated or Matrigel-coated filters, respectively, relative to control-transfectants. Asterisks (*), statistical analysis with the Mann-Whitney *U* test.

The expression profile of *miR-655* was compared with that of each of seven typical EMT-related genes (*CDH1/E-cadherin*, *miR-141*, *-200a*, *-200b*, *-200c*, *-205*, and *VIM*) in a panel of 23 pancreatic cancer cell lines and a breast cancer cell line, MDA-MB-231 (Fig. 2A and Fig. S2). We noticed a consistent positive correlation among expression profiles of *miR-200* family members and a slight correlation between *CDH1/E-cadherin* and *miR-200* family members. Although no correlation between the expression pattern of *miR-655* and that of any of these marker genes was found in this panel, these cell lines all showed lower expression of *miR-655* than the *miR-200* family and *miR-205*, as compared with a normal pancreas. Marked down regulation of *miR-655* expression was also observed in 97.7% (42/43) of ESCC and 94.7% (18/19) of OSCC cell lines (<0.5-fold expression) (Fig. S3A and B). Moreover, the expression of endogenous *miR-655* was higher in MCF7 and MCF10A (human breast epithelial cells) than MDA-MB-231, suggesting that downregulation of *miR-655* might contribute to phenotypic stabilization of mesenchymal feature in MDA-MB-231 cell line (Fig. S3C).

To confirm the EMT-suppressive effects of miR-*655* on mesenchymal-like pancreatic or breast cancer cells having phenotypic plasticity at EMT/MET, we ectopically introduced 10 nM of synthetic dsRNA mimicking mature *miR-655* into Panc1, KP1N, KP4-4 and MDA-MB-231 cells. The Panc1 and KP1N cell lines are *miR-655*-high expressers, while the KP4-4 and MDA-MB-231 cell lines are *miR-655*-low expressers (Fig. 2A). However, in all four cell lines 96 hours after transfection with *miR-655*, a morphological shift toward the epithelial phenotype was induced (Fig. 2B) consistent with an upregulation of *CDH1/E-cadherin* expression at the mRNA and protein levels (Fig. 2C). In addition, we confirmed that ectopic expression of *miR-655* increased *CDH1/E-cadherin* expression at the mRNA and protein levels in an ESCC cell line, TE8, and an OSCC cell line, HSC2, (Fig. S4), although a morphological shift toward the epithelial phenotype in these cell lines was not observed (data not shown). These EMT-suppressive effects of miR-*655* were observed in a *miR-200* family-independent manner (data not shown). To take into consideration off-target effects of dsRNAs, these EMT-suppressive effects of *miR-655* were also confirmed using two kinds of dsRNAs purchased from independent companies (Fig. S5). Notably, effects of overexpression of exogenous *miR-655* on cell growth were not constant in these cell lines (Fig. 2D), whereas the number of cells that migrated through the uncoated or Matrigel-coated membranes in cell migration or invasion assays, respectively, was significantly decreased in all *miR-655*-transfectants compared with their control counterparts (Fig. 2E and 2F). These results suggest that *miR-655* may suppress EMT in mesenchymal-like cancer cells.

### Expression Analysis of *miR-655* in Primary ESCC and OSCC Cases

**Figure 3:**
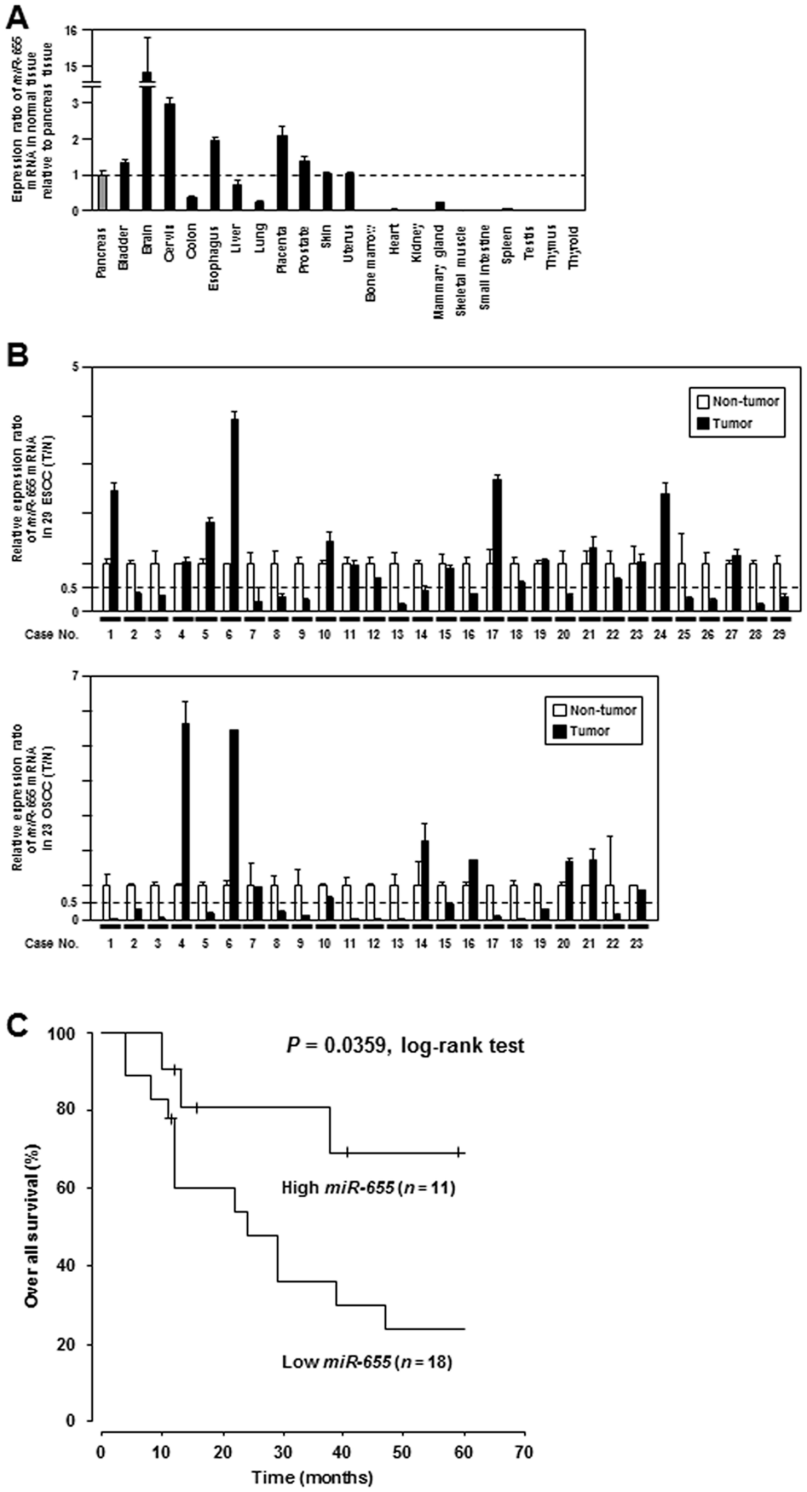
Expression analysis of *miR-655* in primary ESCC and OSCC cases. ***A***, TaqMan real-time RT-PCR analysis of endogenous *miR-655* in 22 normal human tissues (Ambion and Clontech). Marked upregulation of *miR-655* expression (>2-fold increase compared with pancreas) was observed in brain, cervix, esophagus and placenta. ***B***, Expression profiles of *miR-655* in a panel of paired tumorous and non-tumorous tissues from primary ESCC and OSCC cases. Bar graphs show the ratio of the expression level in tumors (T) to those in their paired normal mucosae (N). ***C***, Kaplan-Meier survival curves for high and low *miR-655* groups based on TaqMan real-time RT-PCR. In univariate analyses of overall and non-recurrent survival with log-rank tests, a high level of *miR-655* expression was significantly associated with a much better survival rate among patients with ESCC (*P* = 0.0359, log-rank test).

We investigated the normal human tissue distribution and tumor expression of endogenous *miR-655* by TaqMan RT-PCR analysis. Among 22 normal tissues, upregulation of *miR-655* expression was observed in brain, cervix, esophagus and placenta (>2-fold increase compared with a normal pancreas) (Fig. 3A). We next examined the expression level of the *miR-655* transcript in primary tumors of ESCC and OSCC, respectively. Expression levels of *miR-655* in tumors as compared with paired non-tumorous mucosae were markedly reduced in 44.8% (13/29) and 60.9% (14/23) of primary ESCC and OSCC cases, respectively (<0.5-fold expression) (Fig. 3B). Furthermore, to evaluate the clinical significance of *miR-655* expression in ESCC, we categorized the patients into two groups based on the mean value: a low *miR-655* group (n = 18) and a high *miR-655* group (n = 11). In Kaplan-Meier survival curves for 29 patients with ESCC expressing higher and lower levels of *miR-655*, univariate analyses of overall and non-recurrent survival with log-rank tests demonstrated a significant association between higher levels of *miR-655* expression and a better survival rate (Fig. 3C, *P* = 0.0359, log-rank test), whereas the *miR-655* expression in each tumor was not associated with clinicopathological features (Table S3). These findings suggest that *miR-655* expression may significantly correlate with prognosis in ESCC. We could not analyze the prognostic significance of *miR-655* expression in OSCC because complete survival data was not included in our clinical data. Finally, we analyzed miRNA-target associations at the mRNA level in ESCC and OSCC primary samples (Fig. S6A), however significant correlations were not found between *miR-655* expression and expression of *ZEB1* or *TGFBR2* transcripts as well as a large number of miRNAs and their targets, indicating that not only *miR-655* but also other unknown molecules including transcription factors might regulate *ZEB1* and *TGFBR2* expression. Similar to result in primary samples, there were no correlations between *miR-655* expression and mRNA or protein expression of these targets in ESCC cell lines because of remarkable reduction of *miR-655* expression in almost ESCC cell lines (Fig. S6B).

### Characterization of *ZEB1* and *TGFBR2* as Novel Direct Targets of *miR-655*

**Figure 4:**
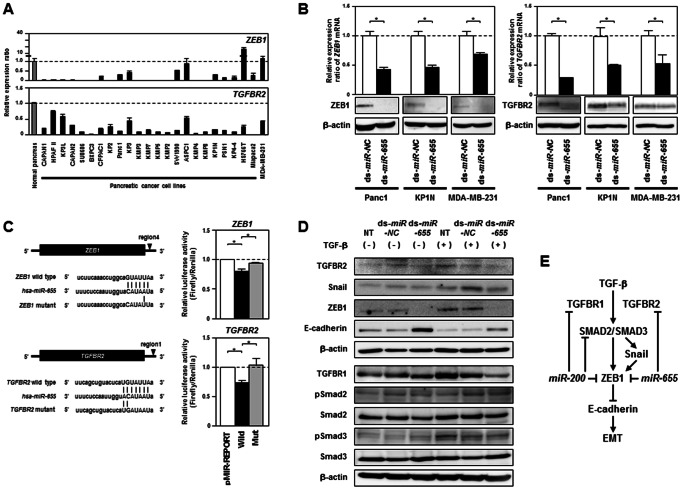
Characterization of *ZEB1* and *TGFBR2* as novel direct targets of *miR-655*. ***A***, Expression analysis for *ZEB1* (***top***) and *TGFBR2* (***bottom***) in a panel of 23 pancreatic cancer cell lines and a breast cancer cell line, MDA-MB-231, using TaqMan real-time RT-PCR. Relative expression levels of transcripts of *ZEB1* and *TGFBR2* were quantified in comparison to *GAPDH* to normalize the initial input of total RNA. Bar graphs show the ratio of the expression level in each cell line to that in a normal pancreas (Ambion). ***B***, TaqMan real-time RT-PCR analysis and Western blot analysis of *ZEB1* (***left***) and *TGFBR2* (***right***) in Panc1, KP1N and MDA-MB-231 cells 96 hours after transfection with 10 nM of dsRNA mimicking *miR-655* (ds-*miR-655*) or control non-specific miRNA (ds-*NC*) (Ambion). ***C***, Confirmation of *ZEB1* and *TGFBR2* as direct targets of *miR-655*. ***Left***, Schema of putative binding sites of *miR-655* in the 3′-UTR region of *ZEB1* and *TGFBR2*. ***Right***, Results of luciferase reporter assays in Panc1 cells 48 hours after cotransfection of pMIR-REPORT luciferase vectors containing wild-type (Wt) or mutated (Mut) 3′-UTR target sites of *ZEB1* or *TGFBR2* for *miR-655*, ds-*miR-655* or ds-*NC*, and pRL-CMV internal control vector. These sites were analyzed using microRNA.org and Target Scan Human 6.2. ***D***, Suppressive effects of ds-*miR-655* on TGF-b-induced EMT in KP1N cells. The results of Western blotting of TGFBR2, Snail, ZEB1, E-cadherin, TGFBR1, Smad2/3 and phosphorylated Smad2/3 in KP1N cells 72 hours after treatment with or without TGF-b (5 ng/ml) and transfection with ds-*miR-655* or ds-*NC*, simultaneously. TGF-b-treated cells were compared with untreated cells. ***E***, Schema of regulation of the ZEB1-E-cadherin axis and TGF-b signaling pathway by *miR-655* through downregulation of *ZEB1* or *TGFBR2* in cancer cells.

We searched the websites microRNA.org (http://www.microrna.org/) and Target Scan Human 6.2 (http://www.targetscan.org/) [39], [40] for direct targets of *miR-655*, and focused on *ZEB1* and *TGFBR2* as potential candidates, respectively. Although expression levels of *ZEB1* and *TGFBR2* tended to be lower in pancreatic cancer cell lines and a breast cancer cell line relative to a normal pancreas (Fig. 4A), transcript and protein levels of these candidate genes were markedly reduced in mesenchymal-like pancreatic or breast cancer cells 96 hours after transfection with dsRNAs mimicking *miR-655* (Fig. 4B). We obtained the same results using other sets of dsRNAs purchased from independent sources (Fig. S7). Moreover, in the luciferase reporter assay with vectors containing the wild type or a mutated 3′-UTR target site of *ZEB1* (region 4) and *TGFBR2* (region 1) downstream of the luciferase reporter gene, we detected statistically significant reductions in luciferase activity in wild type constructs, but not in mutant constructs (Fig. 4C and Fig. S8A and B), indicating that *ZEB1* and *TGFBR2* were novel direct targets of *miR-655*. We confirmed that the treatment with TGF-b could induce EMT accompanied by the upregulation of *TGFBR2*, TGFBR1, *SNAI1/Snail*, *ZEB1*, and *PAI-1* and the downregulation of *CDH1/E-cadherin* at mRNA and protein levels in KP1N cells. Furthermore, overexpression of exogenous *miR-655* significantly inhibited the upregulation and downregulation of these EMT-regulatory genes in KP1N cells treated with and without TGF-b, respectively (Fig. 4D and Fig. S9), whereas *miR-655* could not change levels of phosphorylated Smad2/3 in these cells. Our findings strongly suggest that the TGF-b-induced EMT can be suppressed by *miR-655*, independently of *miR-200* family members, through translational inhibition of *ZEB1* and *TGFBR2* in cancer cells (Fig. 4E).

## Discussion

EMT plays a crucial role in many stages not only in embryonic development but also in cancer progression [3], [6]. Cancer cells undergoing EMT are endowed with more aggressive phenotypes, such as mesenchymal and stem cell-like features, resulting in the acquisition of malignant properties, such as invasion, metastasis, recurrence, and drug resistance [4], [5], [41]. The evidence for EMT, including our own [32], [35], led us to consider that the development of EMT inhibitors might provide opportunities for both prevention and treatment of cancer. Therefore, to identify EMT-suppressive miRNAs, we performed here the function-based screening of 470 dsRNAs mimicking mature human miRNAs using mesenchymal-like cancer cells, Panc1.

Over the last few years, we have focused on miRNAs as key post-transcriptional regulators of gene expression and previously identified four tumor-suppressive miRNAs (TS-miRNAs) directly targeting oncogenic genes in oral squamous cell carcinoma (OSCC) and hepatocellular carcinoma (HCC) using expression-based and DNA methylation-based screening, respectively [15], [33], [42]. In recent studies, we successfully performed function-based screening with a cell proliferation assay for 327 synthetic miRNAs and identified two TS-miRNAs directly targeting *Rictor* in OSCC and endometrial cancer (EC) [15], [26], [27]. In the present study, a unique cell-based reporter system for investigating *CDH1/E-cadherin*-promoter activity in the Panc1 cell line was established and used in the function-based screening of EMT-suppressive miRNAs. In this system, the *CDH1/E-cadherin*-promoter was employed to monitor MET, because the transcription of *CDH1/E-cadherin* is known to be repressed during EMT and activated during MET [26]. Panc1 has already been used as an *in vitro* experimental model for assessing induction of EMT [35], [36]. Since Panc1 was also confirmed to be a mesenchymal-like cell line with phenotypic plasticity at EMT/MET [35], we used it in the establishment of our cell-based reporter system. Actually, *miR-200a*, *-200b* and *-200c* were discovered as EMT-suppressive miRNAs in previous studies, emerged at the top of a list of results of screening, suggesting the present cell-based reporter assay to be a powerful tool for high-throughput function-based screening of miRNAs, siRNAs and chemical compounds having EMT-suppressive effects.

Here, we successfully identified *miR-655* for the first time as a novel EMT-suppressive miRNA through function-based screening. This miRNA had mostly remained uncharacterized in the field of cancer research. The *miR-655* gene is located within a non-coding region at 14q32.31, which harbors 50 intergenic miRNA genes within a limited region of 198 kb. Although notable copy number aberrations were not detected at 14q32.31 by our aCGH analyses using a panel of pancreatic cancer [28], ESCC [29] and OSCC [30], [31] cell lines, the expression of *miR-134* and *miR-370* located at this locus was described to be significantly lower in gastrointestinal stromal tumors (GISTs) with 14q loss and also in GISTs with tumour progression [43]. In the present study, the expression of *miR-655* was largely downregulated in a panel of pancreatic cancer, ESCC and OSCC cell lines, and a breast cancer cell line, MDA-MB-231. Moreover, we found a significant correlation between higher levels of *miR-655* expression and a better survival rate in patients with ESCC, suggesting *miR-655* expression to be a promising prognostic marker for ESCC.

In the present study, *ZEB1* and *TGFBR2* were identified as direct targets of *miR-655*. These targets have been revealed to be major components of TGF-b signaling pathways and to induce EMT through repression of *CDH1/E-cadherin* [17], [19], [44]. *ZEB1* was identified first as a strong predictor of poor survival and distant metastasis in colorectal adenocarcinoma, breast cancer [45], [46], and lung adenocarcinoma [47]. High *TGFBR2* expression was also correlated with a shorter overall survival in estrogen receptor-negative breast cancer [48]. *miR-520c*, *miR-373*, and *miR-211* were described as miRNAs targeting *TGFBR2* and contributing to the induction of MET [25], [49] although these three miRNAs were not identified as prime candidates of EMT-suppressive miRNA in our screening. The *miR-200* family members are typical EMT-suppressive miRNAs targeting several components of TGF-b signaling pathways, including the *miR-200*-ZEB1-E-cadherin axis, which is crucial in EMT and was described to be deregulated in mesenchymal-like cancer cells [17], [18], [20]. Although the *miR-200* family and *miR-205*, like *miR-655*, target *ZEB1*, their biological functions were found to differ from those of *miR-655*. First, besides *ZEB1*, *TGFBR2* was characterized as a direct target of *miR-655* in our study, but not the *miR-200* family and *miR-205*. Second, our studies past and present, have showed the mesenchymal-specific downregulation of *miR-200* expression in a panel of OSCC [35] and pancreatic cancer cell lines, respectively, but not *miR-205* and *miR-655* expression. These differences between *miR-655* and *miR-200* family members indicate the biological function of each EMT-suppressive miRNA in physiological and pathophysiological processes, including EMT/MET. In addition, several components of TGF-b signaling pathways, TGFBR2, Snail and ZEB1, were reduced directly or indirectly by overexpression of *miR-655* in cancer cells treated with or without TGF-b. Recent studies have demonstrated that a miRNA significantly decreased signal output over time, by reducing the concentration of several components in a signaling cascade [50], [51]. These results strongly support our findings that a single EMT-suppressive miRNA may target several EMT-inducible components of a specific signaling pathway and coordinate their expression. On the other hand, overexpression of *miR-655*, as well as the *miR-200* family, induced significant morphologic changes and inhibited cell migration and invasion in 3 pancreatic cancer cell lines and a breast cancer cell line, MDA-MB-231. These observations suggest the EMT-suppressive effects of *miR-655* to be essential for cancer progression.

In conclusion, we established a unique cell-based reporter system for monitoring the promoter activity of *CDH1/E-cadherin*. By using the system for the first time we identified *miR-655* as a novel EMT-suppressive miRNA, the biological meaning of which was different from that of the *miR-200* family. Overexpression of *miR-655* remarkably increased E-cadherin expression and suppressed cell motility in several cancer cell lines, clearly indicating that this miRNA is a strong suppressor of EMT. In ESCC, *miR-655* expression demonstrated a significant association with a better prognosis. Furthermore, *ZEB1* and *TGFBR2*, which are cardinal components of the TGF-b signaling pathway, were characterized as direct targets of *miR-655*. Our results suggest the potential of the EMT-suppressor *miR-655* targeting *ZEB1* and *TGFBR2* as a prognostic marker and therapeutic agent for cancer.

## Supporting Information

Figure S1 Fluorescence micrographs of a stable Panc1 clone 96 hours after transient transfection with dsRNA in functional-based screening using Pre-miR™ miRNA Precursor Library - Human V3 (Ambion). The Panc1 clone was established by transfection with a reporter construct containing a promoter sequence of *CDH1/E-cadherin* in the 5′ upstream region of the *ZsGreen1* reporter gene and cloning using limiting dilution (see Fig. 1A, and 1B). Each dsRNA was transfected individually into the clone. These 17 miRNA genes were selected as candidates for EMT-suppressive miRNAs in functional-based screening (see Table 1, Fig. 1C, and Table S2).
(PPT)

Figure S2 Expression profiles of known EMT-related genes, *miR-141*, *-200a*, *-200b*, *-200c*, *-205* and *VIM*, in a panel of 23 pancreatic cancer cell lines and a breast cancer cell line, MDA-MB-231 (see Fig. 2A and 4A). Bar graphs show the ratio of the expression level in these cell lines to that in a normal pancreas tissue (Ambion) by TaqMan real-time RT-PCR analysis.
(PPT)

Figure S3 Expression profiles of *miR-655* in a panel of 43 ESCC cell lines. (***A***) and 18 OSCC cell lines (***B***). Bar graphs show the ratio of the expression level in ESCC and OSCC cell lines to that in normal esophageal tissue (Ambion). ***C***, Expression profiles of *miR-655* in normal esophagus and mammary gland, MCF7, MCF10A (human breast epithelial cells) and MDA-MB-231.
(PPT)

Figure S4 TaqMan real-time RT-PCR analysis (***Upper***) and Western blot (***Lower***) analysis of mRNA and protein levels of *CDH1/E-cadherin*, respectively, in TE8 and HSC2 cells 96 hours after transfection of 10 nM of ds-*NC* or ds-*miR-655*.
(PPT)

Figure S5 TaqMan real-time RT-PCR analysis (***Upper***) and Western blot (***Lower***) analysis for *CDH1/E-cadherin* in Panc1, KP1N and MDA-MB-231 cells 96 hours after transfection of 10 nM of ds-*NC* or ds-*miR-655* (Thermo Scientific Dharmacon).
(PPT)

Figure S6 ***A***, The correlations between *miR-655* and *ZEB1/TGFBR2* on mRNA levels in ESCC/OSCC primary samples. ***B***, The correlations between *miR-655* and ZEB1/TGFBR2 on mRNA and protein levels. The quantification of each protein band in the result of Western blotting was done using LAS-3000 with MultiGauge software (GE Healthcare, Tokyo, Japan). Pearson’s test was performed to determine the degree of correlation between two variables.
(PPT)

Figure S7 TaqMan real-time RT-PCR analysis (***Upper***) and Western blot (***Lower***) analysis for *ZEB1* (***left***) and *TGFBR2* (***right***) in Panc1, KP1N and MDA-MB-231 cells 96 hours after transfection of 10 nM of ds-*NC* or ds-*miR-655* (Thermo Scientific Dharmacon).
(PPT)

Figure S8 ***A***, Complementary miR-655 seed sequence and PCR region in the 3′UTR of *ZEB1* (***Upper***) and *TGFBR2* (***Lower***). These sites were analyzed using microRNA.org and Target Scan Human 6.2. ***B***, Results of luciferase reporter assays in Panc1 cells 48 hours after cotransfection of pMIR-REPORT luciferase vectors containing wild-type of *ZEB1* or *TGFBR2* for *miR-655*, ds-*miR-655* or ds-*NC*, and the pRL-CMV internal control vector. Asterisks (*), statistical analysis with the Mann-Whitney *U* test.
(PPT)

Figure S9 TaqMan real-time RT-PCR analysis for *CDH1/E-cadherin* (***left***) and *PAI-1* (***right***) in KP1N cells 96 hours after transfection of 10 nM of ds-*NC* or ds-*miR-655* (Ambion). Cells were analyzed 72 hours after treatment with or without TGF-b (5 ng/ml) and transfection with ds-*miR-655* or ds-*NC*, simultaneously.
(PPT)

Table S1 Primers used in this study.
(XLS)

Table S2 Summary of functional-based screening using 470 dsRNAs mimicking mature miRNAs (Pre-miR™ miRNA Precursor Library - Human V3, Ambion) and a stable Panc1 clone transfected with a reporter construct containing a promoter sequence of *CDH1/E-cadherin* in the 5′ upstream region of the *ZsGreen1* reporter gene.
(XLS)

Table S3 Correlation between clinicopathological characteristics and status of *miR-655* expression in primary ESCC cases.
(XLS)

## References

[1] Greenburg G, Hay ED (1982) Epithelia suspended in collagen gels can lose polarity and express characteristics of migrating mesenchymal cells. J Cell Biol 95: 333–339. 7142291 10.1083/jcb.95.1.333 PMC2112361

[2] Thiery JP (2002) Epithelial-mesenchymal transitions in tumour progression. Nat Rev Cancer 2: 442–454. 12189386 10.1038/nrc822

[3] Thiery JP, Acloque H, Huang RY, Nieto MA (2009) Epithelial-mesenchymal transitions in development and disease. Cell 139: 871–890. 19945376 10.1016/j.cell.2009.11.007

[4] Iwatsuki M, Mimori K, Yokobori T, Ishi H, Beppu T, et al (2010) Epithelial-mesenchymal transition in cancer development and its clinical significance. Cancer Sci 101: 293–299. 19961486 10.1111/j.1349-7006.2009.01419.x PMC11159985

[5] Micalizzi DS, Farabaugh SM, Ford HL (2010) Epithelial-mesenchymal transition in cancer: parallels between normal development and tumor progression. J Mammary Gland Biol Neoplasia 15: 117–134. 20490631 10.1007/s10911-010-9178-9 PMC2886089

[6] Kalluri R, Weinberg RA (2009) The basics of epithelial-mesenchymal transition. J Clin Invest 119: 1420–1428. 19487818 10.1172/JCI39104 PMC2689101

[7] Zhang J, Ma L (2012) MicroRNA control of epithelial-mesenchymal transition and metastasis. Cancer Metastasis Rev. 10.1007/s10555-012-9368-6 PMC3686549 22684369

[8] Li Y, VandenBoom TG, Kong D, Wang Z, Ali S, et al (2009) Up-regulation of miR-200 and let-7 by natural agents leads to the reversal of epithelial-to-mesenchymal transition in gemcitabine-resistant pancreatic cancer cells. Cancer Res 69: 6704–6712. 19654291 10.1158/0008-5472.CAN-09-1298 PMC2727571

[9] Sabbah M, Emami S, Redeuilh G, Julien S, Prévost G, et al (2008) Molecular signature and therapeutic perspective of the epithelial-to-mesenchymal transitions in epithelial cancers. Drug Resist Updat 11: 123–151. 18718806 10.1016/j.drup.2008.07.001

[10] Ambros V (2004) The functions of animal microRNAs. Nature 431: 350–355. 15372042 10.1038/nature02871

[11] Bartel DP (2004) MicroRNAs: genomics, biogenesis, mechanism, and function. Cell 116: 281–297. 14744438 10.1016/s0092-8674(04)00045-5

[12] He L, Hannon GJ (2004) MicroRNAs: small RNAs with a big role in gene regulation. Nat Rev Genet 5: 522–531. 15211354 10.1038/nrg1379

[13] Esquela-Kerscher A, Slack FJ (2006) Oncomirs - microRNAs with a role in cancer. Nat Rev Cancer 6: 259–269. 16557279 10.1038/nrc1840

[14] Osada H, Takahashi T (2007) MicroRNAs in biological processes and carcinogenesis. Carcinogenesis 28: 2–12. 17028302 10.1093/carcin/bgl185

[15] Kozaki K, Inazawa J (2012) Tumor-suppressive microRNA silenced by tumor-specific DNA hypermethylation in cancer cells. Cancer Sci 103: 837–845. 22320679 10.1111/j.1349-7006.2012.02236.x PMC7659391

[16] Bader AG, Brown D, Winkler M (2012) The promise of microRNA replacement therapy. Cancer Res 70 7027–30. 10.1158/0008-5472.CAN-10-2010 PMC2940943 20807816

[17] Gregory PA, Bert AG, Paterson EL, Barry SC, Tsykin A, et al (2008) The miR-200 family and miR-205 regulate epithelial to mesenchymal transition by targeting ZEB1 and SIP1. Nat Cell Biol 10: 593–601. 18376396 10.1038/ncb1722

[18] Bracken CP, Gregory PA, Kolesnikoff N, Bert AG, Wang J, et al (2008) A double-negative feedback loop between ZEB1-SIP1 and the microRNA-200 family regulates epithelial-mesenchymal transition. Cancer Res 68: 7846–7854. 18829540 10.1158/0008-5472.CAN-08-1942

[19] Gregory PA, Bracken CP, Smith E, Bert AG, Wright JA, et al (2011) An autocrine TGF-beta/ZEB/miR-200 signaling network regulates establishment and maintenance of epithelial-mesenchymal transition. Mol Biol Cell 22: 1686–1698. 21411626 10.1091/mbc.E11-02-0103 PMC3093321

[20] Park SM, Gaur AB, Lengyel E, Peter ME (2008) The miR-200 family determines the epithelial phenotype of cancer cells by targeting the E-cadherin repressors ZEB1 and ZEB2. Genes Dev 22: 894–907. 18381893 10.1101/gad.1640608 PMC2279201

[21] Burk U, Schubert J, Wellner U, Schmalhofer O, Vincan E, et al (2008) A reciprocal repression between ZEB1 and members of the miR-200 family promotes EMT and invasion in cancer cells. EMBO Rep 9: 582–589. 18483486 10.1038/embor.2008.74 PMC2396950

[22] Brabletz S, Bajdak K, Meidhof S, Burk U, Niedermann G, et al (2011) The ZEB1/miR-200 feedback loop controls Notch signalling in cancer cells. EMBO J 30: 770–782. 21224848 10.1038/emboj.2010.349 PMC3041948

[23] Mani SA, Guo W, Liao MJ, Eaton EN, Ayyanan A, et al (2008) The epithelial-mesenchymal transition generates cells with properties of stem cells.Cell. 133: 704–715. 10.1016/j.cell.2008.03.027 PMC2728032 18485877

[24] Nakano H, Miyazawa T, Kinoshita K, Yamada Y, Yoshida T (2010) Functional screening identifies a microRNA, miR-491 that induces apoptosis by targeting Bcl-X(L) in colorectal cancer cells. Int J Cancer 127: 1072–1080. 20039318 10.1002/ijc.25143

[25] Levy C, Khaled M, Iliopoulos D, Janas MM, Schubert S, et al (2010) Intronic miR-211 assumes the tumor suppressive function of its host gene in melanoma. Mol Cell 40: 841–849. 21109473 10.1016/j.molcel.2010.11.020 PMC3004467

[26] Tsuruta T, Kozaki K, Uesugi A, Furuta M, Hirasawa A, et al (2011) miR-152 is a tumor suppressor microRNA that is silenced by DNA hypermethylation in endometrial cancer. Cancer Res 71: 6450–6462. 21868754 10.1158/0008-5472.CAN-11-0364

[27] Uesugi A, Kozaki K, Tsuruta T, Furuta M, Morita K, et al (2011) The tumor suppressive microRNA miR-218 targets the mTOR component Rictor and inhibits AKT phosphorylation in oral cancer. Cancer Res 71: 5765–5778. 21795477 10.1158/0008-5472.CAN-11-0368

[28] Suzuki A, Shibata T, Shimada Y, Murakami Y, Horii A, et al (2008) Identification of SMURF1 as a possible target for 7q21.3–22.1 amplification detected in a pancreatic cancer cell line by in-house array-based comparative genomic hybridization. Cancer Sci 99: 986–994. 18380791 10.1111/j.1349-7006.2008.00779.x PMC11158928

[29] Haruki S, Imoto I, Kozaki K, Matsui T, Kawachi H, et al (2010) Frequent silencing of protocadherin 17, a candidate tumour suppressor for esophageal squamous cell carcinoma. Carcinogenesis 31: 1027–1036. 20200074 10.1093/carcin/bgq053

[30] Suzuki E, Imoto I, Pimkhaokham A, Nakagawa T, Kamata N, et al (2007) PRTFDC1, a possible tumor-suppressor gene, is frequently silenced in oral squamous-cell carcinomas by aberrant promoter hypermethylation. Oncogene 26: 7921–7932. 17599052 10.1038/sj.onc.1210589

[31] Nakamura E, Kozaki K, Tsuda H, Suzuki E, Pimkhaokham A, et al (2008) Frequent silencing of a putative tumor suppressor gene melatonin receptor 1 A (MTNR1A) in oral squamous-cell carcinoma. Cancer Sci 99: 1390–1400. 18452558 10.1111/j.1349-7006.2008.00838.x PMC11158686

[32] Ono H, Imoto I, Kozaki K, Tsuda H, Matsui T, et al.. (2012) SIX1 promotes epithelial-mesenchymal transition in colorectal cancer through ZEB1 activation. Oncogene. 10.1038/onc.2011.646 22286765

[33] Kozaki K, Imoto I, Mogi S, Omura K, Inazawa J (2008) Exploration of tumor-suppressive microRNAs silenced by DNA hypermethylation in oral cancer. Cancer Res 68: 2094–2105. 18381414 10.1158/0008-5472.CAN-07-5194

[34] Berx G, van Roy F (2009) Involvement of members of the cadherin superfamily in cancer. Cold Spring Harb Perspect Biol 1: a003129. 20457567 10.1101/cshperspect.a003129 PMC2882122

[35] Kurasawa Y, Kozaki K, Pimkhaokham A, Muramatsu T, Ono H, et al (2012) Stabilization of phenotypic plasticity through mesenchymal-specific DNA hypermethylation in cancer cells. Oncogene 31: 1963–1974. 21874048 10.1038/onc.2011.373

[36] Yu J, Ohuchida K, Mizumoto K, Sato N, Kayashima T, et al (2010) MicroRNA, hsa-miR-200c, is an independent prognostic factor in pancreatic cancer and its upregulation inhibits pancreatic cancer invasion but increases cell proliferation. Mol Cancer 9: 169. 20579395 10.1186/1476-4598-9-169 PMC2909980

[37] Wellner U, Schubert J, Burk UC, Schmalhofer O, Zhu F, et al (2009) The EMT-activator ZEB1 promotes tumorigenicity by repressing stemness-inhibiting microRNAs. Nat Cell Biol 11: 1487–1495. 19935649 10.1038/ncb1998

[38] Korpal M, Lee ES, Hu G, Kang Y (2008) The miR-200 family inhibits epithelial-mesenchymal transition and cancer cell migration by direct targeting of E-cadherin transcriptional repressors ZEB1 and ZEB2. J Biol Chem 283: 14910–14914. 18411277 10.1074/jbc.C800074200 PMC3258899

[39] Betel D, Wilson M, Gabow A, Marks DS, Sander C (2008) The microRNA.org resource: targets and expression. Nucleic Acids Res 36: D149–153. 18158296 10.1093/nar/gkm995 PMC2238905

[40] Betel D, Koppal A, Agius P, Sander C, Leslie C (2010) Comprehensive modeling of microRNA targets predicts functional non-conserved and non-canonical sites. Genome Biol 11: R90. 20799968 10.1186/gb-2010-11-8-r90 PMC2945792

[41] Singh A, Settleman J (2010) EMT, cancer stem cells and drug resistance: an emerging axis of evil in the war on cancer. Oncogene 29: 4741–4751. 20531305 10.1038/onc.2010.215 PMC3176718

[42] Furuta M, Kozaki KI, Tanaka S, Arii S, Imoto I, et al (2010) miR-124 and miR-203 are epigenetically silenced tumor-suppressive microRNAs in hepatocellular carcinoma. Carcinogenesis 31: 766–776. 19843643 10.1093/carcin/bgp250

[43] Haller F, von Heydebreck A, Zhang JD, Gunawan B, Langer C, et al (2010) Localization- and mutation-dependent microRNA (miRNA) expression signatures in gastrointestinal stromal tumours (GISTs), with a cluster of co-expressed miRNAs located at 14q32.31. J Pathol 220: 71–86. 19768731 10.1002/path.2610

[44] Li L, Shi JY, Zhu GQ, Shi B (2012) MiR-17–92 cluster regulates cell proliferation and collagen synthesis by targeting TGFB pathway in mouse palatal mesenchymal cells. J Cell Biochem 113: 1235–1244. 22095742 10.1002/jcb.23457

[45] Spaderna S, Schmalhofer O, Hlubek F, Berx G, Eger A, et al (2006) A transient, EMT-linked loss of basement membranes indicates metastasis and poor survival in colorectal cancer. Gastroenterology 131: 830–840. 16952552 10.1053/j.gastro.2006.06.016

[46] Spaderna S, Schmalhofer O, Wahlbuhl M, Dimmler A, Bauer K, et al (2008) The transcriptional repressor ZEB1 promotes metastasis and loss of cell polarity in cancer. Cancer Res 68: 537–544. 18199550 10.1158/0008-5472.CAN-07-5682

[47] Liu Y, Yan X, Liu N, Zhou J, Liu J, et al.. (2012) Lentivirus-delivered ZEB-1 small interfering RNA inhibits lung adenocarcinoma cell growth in vitro and in vivo. J Cancer Res Clin Oncol. 10.1007/s00432-012-1206-2 PMC11824471 22481253

[48] Buck MB, Fritz P, Dippon J, Zugmaier G, Knabbe C (2004) Prognostic significance of transforming growth factor beta receptor II in estrogen receptor-negative breast cancer patients. Clin Cancer Res 10: 491–498. 14760070 10.1158/1078-0432.ccr-0320-03

[49] Keklikoglou I, Koerner C, Schmidt C, Zhang JD, Heckmann D, et al.. (2011) MicroRNA-520/373 family functions as a tumor suppressor in estrogen receptor negative breast cancer by targeting NF-κB and TGF-b signaling pathways. Oncogene. 10.1038/onc.2011.571 22158050

[50] Linsley PS, Schelter J, Burchard J, Kibukawa M, Martin MM, et al (2007) Transcripts targeted by the microRNA-16 family cooperatively regulate cell cycle progression. Mol Cell Biol 27: 2240–2252. 17242205 10.1128/MCB.02005-06 PMC1820501

[51] Ebert MS, Sharp PA (2012) Roles for microRNAs in conferring robustness to biological processes. Cell 149: 515–524. 22541426 10.1016/j.cell.2012.04.005 PMC3351105

