# Supplementary material for: miR-655 Is an EMT-Suppressive MicroRNA Targeting ZEB1 and TGFBR2
Source: PLoS One. 2013 May 14;8(5):e62757. doi: 10.1371/journal.pone.0062757 (PMC3653886; doi:10.1371/journal.pone.0062757)
Supplement: Figure S1 — Fluorescence micrographs of a stable Panc1 clone 96 hours after transient transfection with dsRNA in functional-based screening using Pre-miR™ miRNA Precursor Library - Human V3 (Ambion). The Panc1 clone was established by transfection with a reporter construct containing a promoter sequence of CDH1/E-cadherin in the 5′ upstream region of the ZsGreen1 reporter gene and cloning using limiting dilution (see Fig. 1A, and 1B). Each dsRNA was transfected individually into the clone. These 17 miRNA genes were selected as candidates for EMT-suppressive miRNAs in functional-based screening (see Table 1, Fig. 1C, and Table S2). (PPT) [file pone.0062757.s001.ppt]

## Slide 1
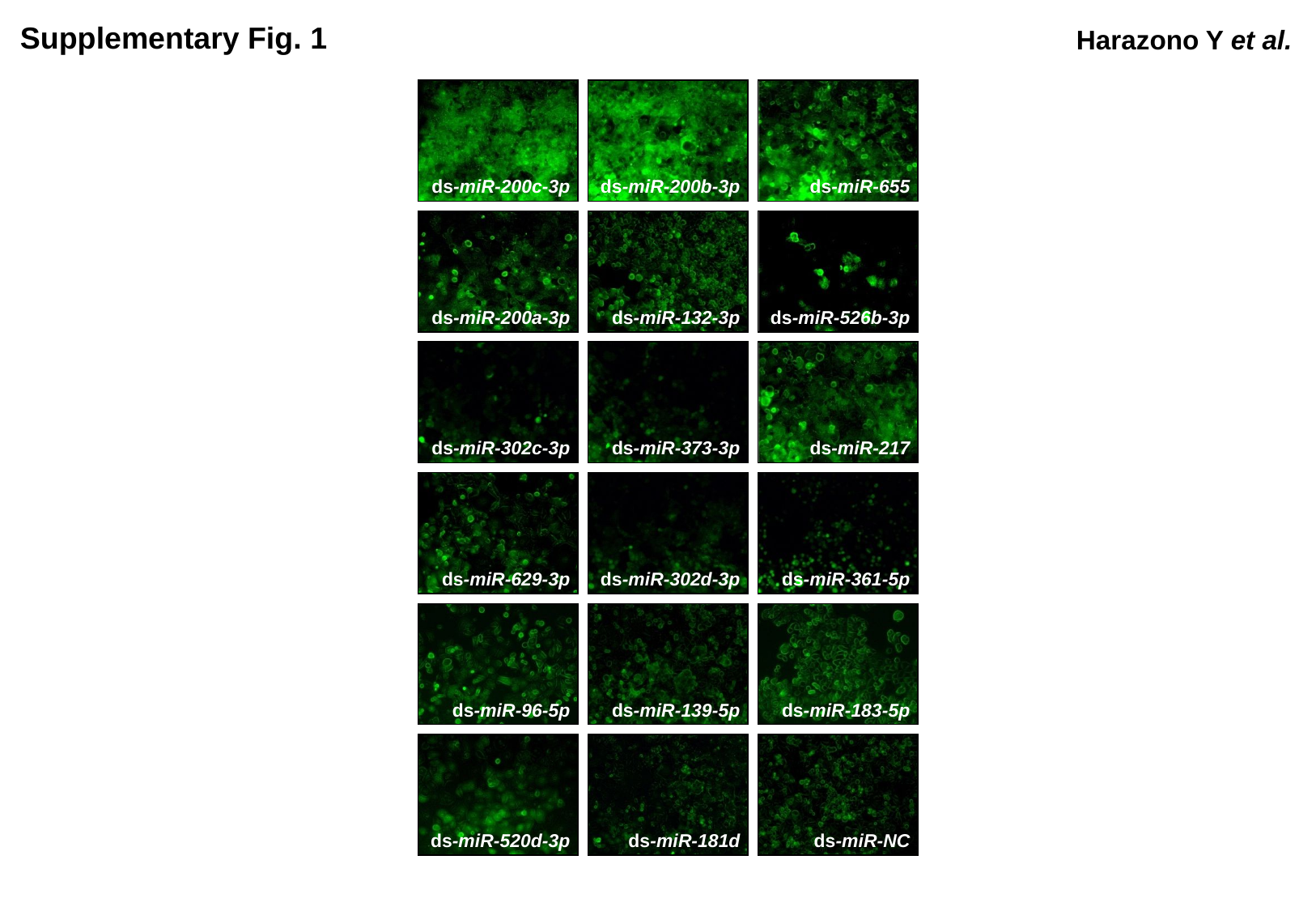

Supplementary Fig. 1
Harazono Y et al.
ds-miR-200c-3p
ds-miR-200b-3p
ds-miR-655
ds-miR-200a-3p
ds-miR-132-3p
ds-miR-526b-3p
ds-miR-302c-3p
ds-miR-373-3p
ds-miR-217
ds-miR-629-3p
ds-miR-302d-3p
ds-miR-361-5p
ds-miR-96-5p
ds-miR-139-5p
ds-miR-183-5p
ds-miR-520d-3p
ds-miR-181d
ds-miR-NC
