# Supplementary material for: miR-655 Is an EMT-Suppressive MicroRNA Targeting ZEB1 and TGFBR2
Source: PLoS One. 2013 May 14;8(5):e62757. doi: 10.1371/journal.pone.0062757 (PMC3653886; doi:10.1371/journal.pone.0062757)
Supplement: Figure S2 — Expression profiles of known EMT-related genes, miR-141, -200a, -200b, -200c, -205 and VIM, in a panel of 23 pancreatic cancer cell lines and a breast cancer cell line, MDA-MB-231 (see Fig. 2A and 4A). Bar graphs show the ratio of the expression level in these cell lines to that in a normal pancreas tissue (Ambion) by TaqMan real-time RT-PCR analysis. (PPT) [file pone.0062757.s002.ppt]

## Slide 1
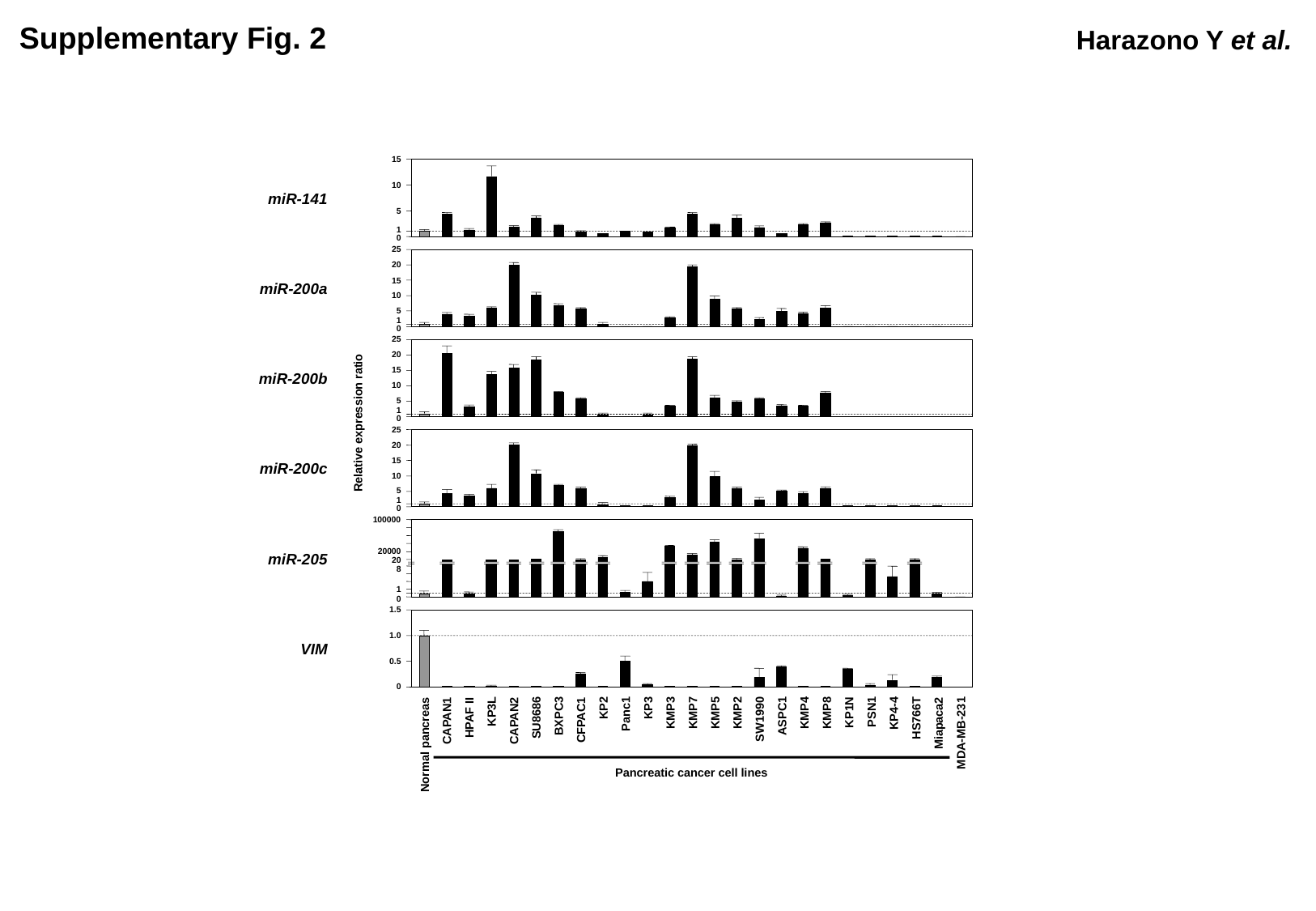

Supplementary Fig. 2
Harazono Y et al.
15
10
miR-141
5
1
0
25
20
15
miR-200a
10
5
1
0
25
20
15
miR-200b
10
5
1
0
Relative expression ratio
25
20
15
miR-200c
10
5
1
0
100000
20000
miR-205
20
8
1
0
1.5
1.0
VIM
0.5
0
KP2
KP3
KP3L
PSN1
KP1N
KMP3
KMP7
KMP5
KMP2
KMP4
KMP8
KP4-4
Panc1
BXPC3
ASPC1
HPAF II
SU8686
HS766T
SW1990
CFPAC1
CAPAN1
CAPAN2
Miapaca2
MDA-MB-231
Normal pancreas
Pancreatic cancer cell lines
