# Supplementary material for: miR-655 Is an EMT-Suppressive MicroRNA Targeting ZEB1 and TGFBR2
Source: PLoS One. 2013 May 14;8(5):e62757. doi: 10.1371/journal.pone.0062757 (PMC3653886; doi:10.1371/journal.pone.0062757)
Supplement: Figure S3 — Expression profiles of miR-655 in a panel of 43 ESCC cell lines. (A) and 18 OSCC cell lines (B). Bar graphs show the ratio of the expression level in ESCC and OSCC cell lines to that in normal esophageal tissue (Ambion). C, Expression profiles of miR-655 in normal esophagus and mammary gland, MCF7, MCF10A (human breast epithelial cells) and MDA-MB-231. (PPT) [file pone.0062757.s003.ppt]

## Slide 1
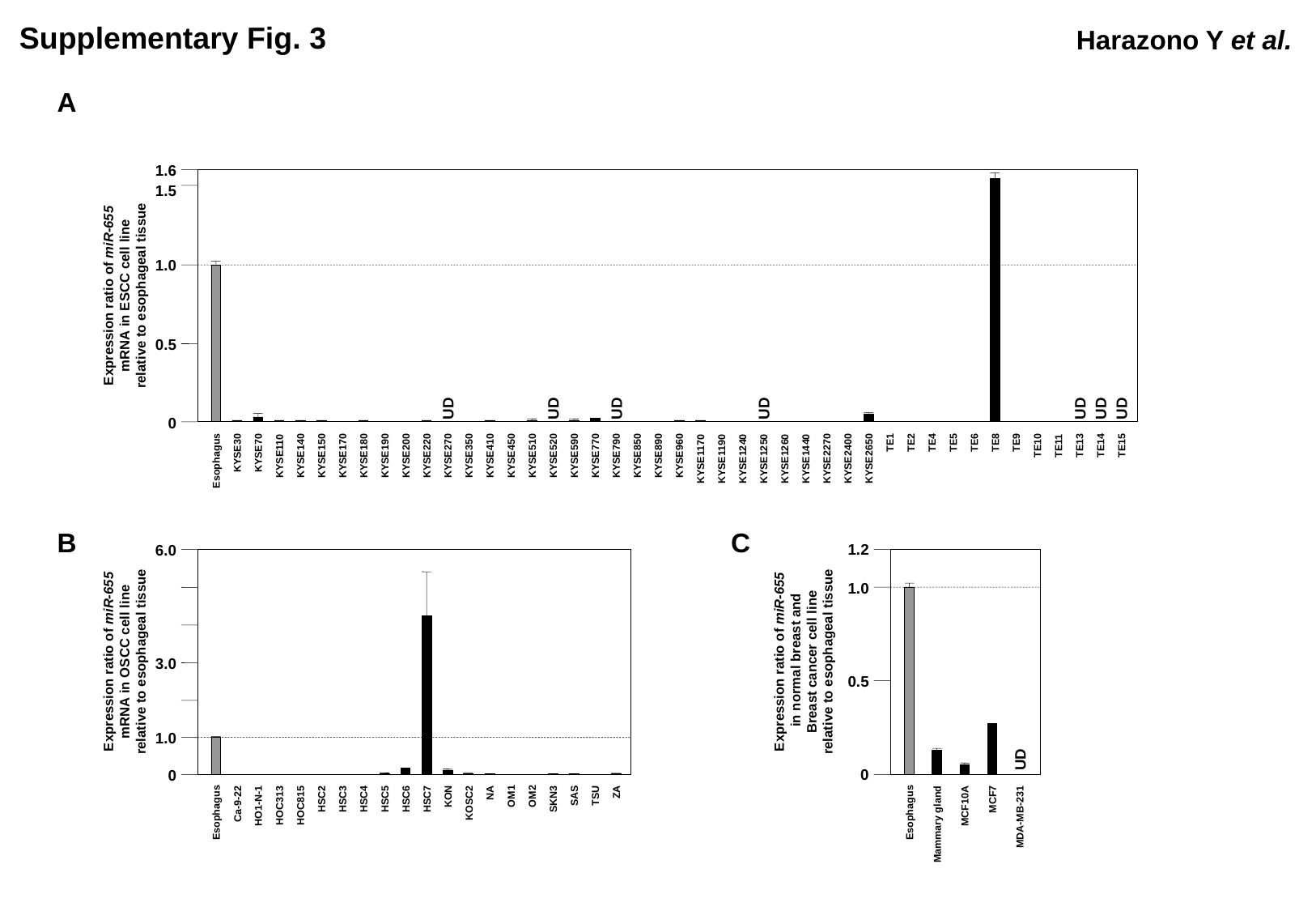

Supplementary Fig. 3
Harazono Y et al.
A
1.6
1.5
1.0
Expression ratio of miR-655
mRNA in ESCC cell line
relative to esophageal tissue
0.5
UD
UD
UD
UD
UD
UD
UD
0
TE1
TE2
TE4
TE5
TE6
TE8
TE9
TE10
TE11
TE13
TE14
TE15
KYSE30
KYSE70
KYSE110
KYSE140
KYSE150
KYSE170
KYSE180
KYSE190
KYSE200
KYSE220
KYSE270
KYSE350
KYSE410
KYSE450
KYSE510
KYSE520
KYSE590
KYSE770
KYSE790
KYSE850
KYSE890
KYSE960
KYSE1170
KYSE1190
KYSE1240
KYSE1250
KYSE1260
KYSE1440
KYSE2270
KYSE2400
KYSE2650
Esophagus
B
C
1.2
6.0
UD
1.0
Expression ratio of miR-655
 in normal breast and
Breast cancer cell line
relative to esophageal tissue
Expression ratio of miR-655
mRNA in OSCC cell line
relative to esophageal tissue
3.0
0.5
1.0
0
0
ZA
NA
TSU
SAS
OM1
OM2
KON
HSC2
HSC3
HSC4
HSC5
HSC6
HSC7
SKN3
MCF7
KOSC2
Ca-9-22
HOC313
HOC815
HO1-N-1
MCF10A
Esophagus
Esophagus
MDA-MB-231
Mammary gland
