# Supplementary material for: miR-655 Is an EMT-Suppressive MicroRNA Targeting ZEB1 and TGFBR2
Source: PLoS One. 2013 May 14;8(5):e62757. doi: 10.1371/journal.pone.0062757 (PMC3653886; doi:10.1371/journal.pone.0062757)
Supplement: Figure S4 — TaqMan real-time RT-PCR analysis (Upper) and Western blot (Lower) analysis of mRNA and protein levels of CDH1/E-cadherin, respectively, in TE8 and HSC2 cells 96 hours after transfection of 10 nM of ds-NC or ds-miR-655. (PPT) [file pone.0062757.s004.ppt]

## Slide 1
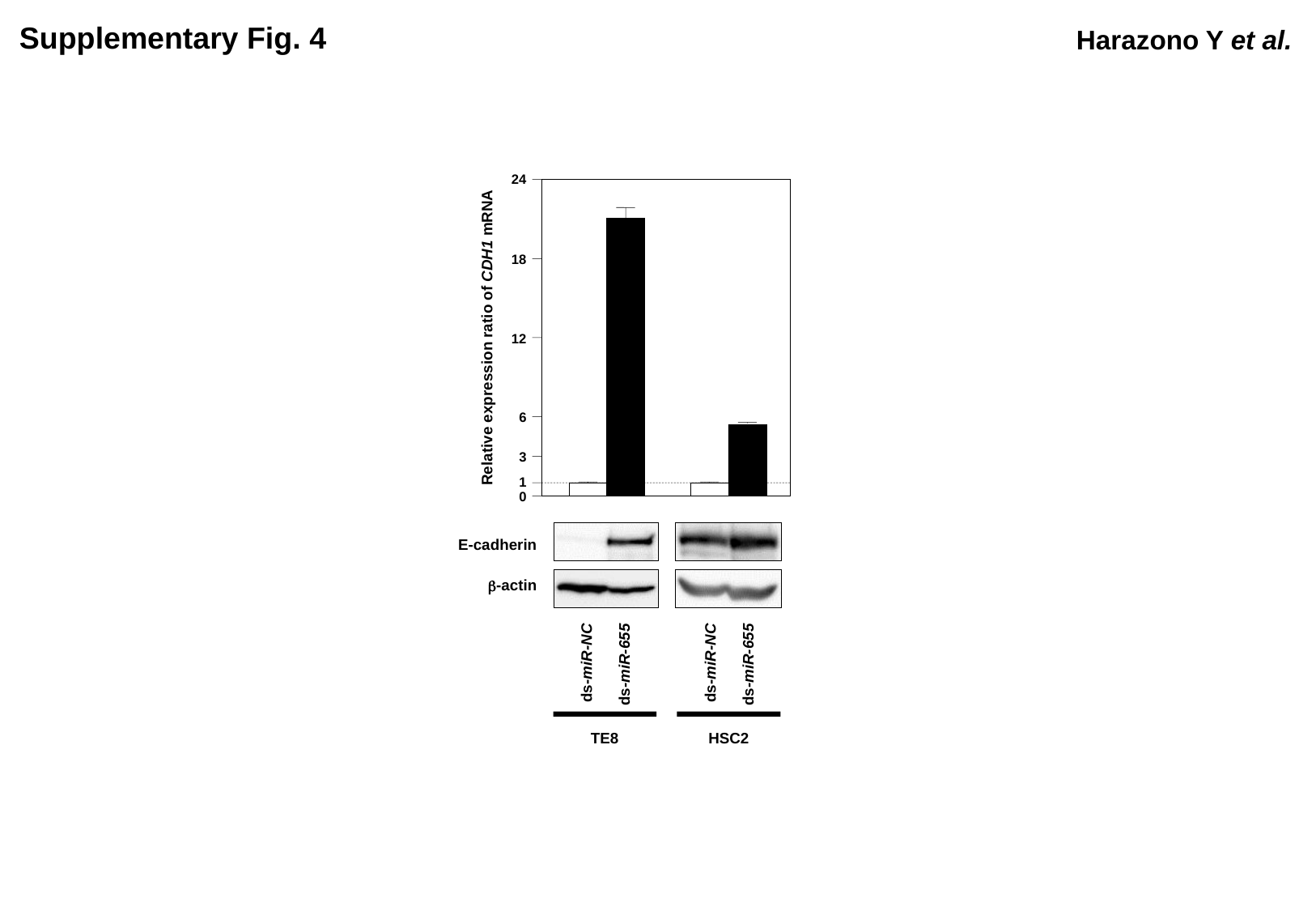

Supplementary Fig. 4
Harazono Y et al.
24
18
Relative expression ratio of CDH1 mRNA
12
6
3
1
0
E-cadherin
β-actin
ds-miR-NC
ds-miR-NC
ds-miR-655
ds-miR-655
TE8
HSC2
