# Supplementary material for: miR-655 Is an EMT-Suppressive MicroRNA Targeting ZEB1 and TGFBR2
Source: PLoS One. 2013 May 14;8(5):e62757. doi: 10.1371/journal.pone.0062757 (PMC3653886; doi:10.1371/journal.pone.0062757)
Supplement: Figure S5 — TaqMan real-time RT-PCR analysis (Upper) and Western blot (Lower) analysis for CDH1/E-cadherin in Panc1, KP1N and MDA-MB-231 cells 96 hours after transfection of 10 nM of ds-NC or ds-miR-655 (Thermo Scientific Dharmacon). (PPT) [file pone.0062757.s005.ppt]

## Slide 1
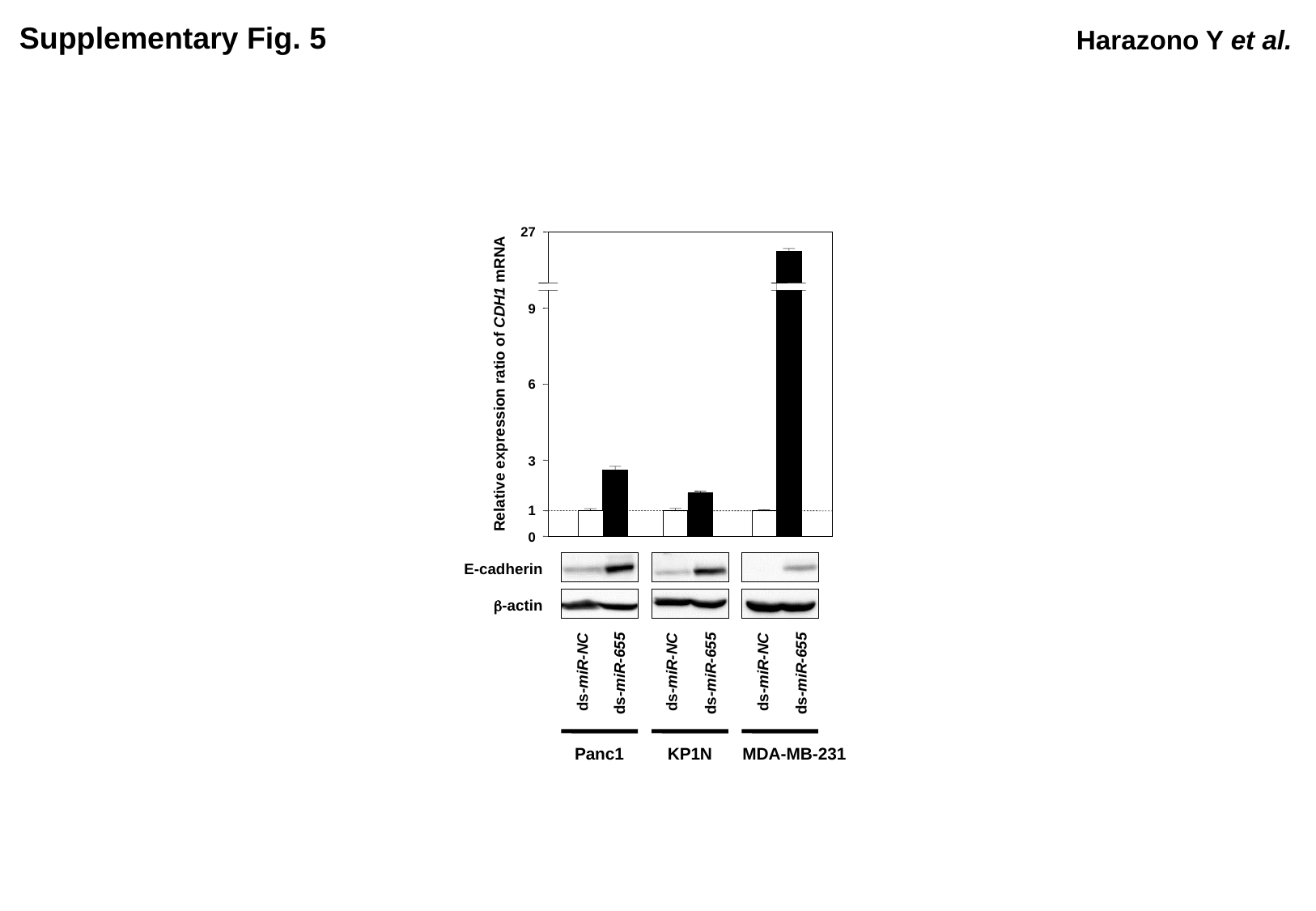

Supplementary Fig. 5
Harazono Y et al.
27
9
Relative expression ratio of CDH1 mRNA
6
3
1
0
E-cadherin
β-actin
ds-miR-NC
ds-miR-NC
ds-miR-NC
ds-miR-655
ds-miR-655
ds-miR-655
Panc1
KP1N
MDA-MB-231
