# Supplementary material for: miR-655 Is an EMT-Suppressive MicroRNA Targeting ZEB1 and TGFBR2
Source: PLoS One. 2013 May 14;8(5):e62757. doi: 10.1371/journal.pone.0062757 (PMC3653886; doi:10.1371/journal.pone.0062757)
Supplement: Figure S6 — A, The correlations between miR-655 and ZEB1/TGFBR2 on mRNA levels in ESCC/OSCC primary samples. B, The correlations between miR-655 and ZEB1/TGFBR2 on mRNA and protein levels. The quantification of each protein band in the result of Western blotting was done using LAS-3000 with MultiGauge software (GE Healthcare, Tokyo, Japan). Pearson’s test was performed to determine the degree of correlation between two variables. (PPT) [file pone.0062757.s006.ppt]

## Slide 1
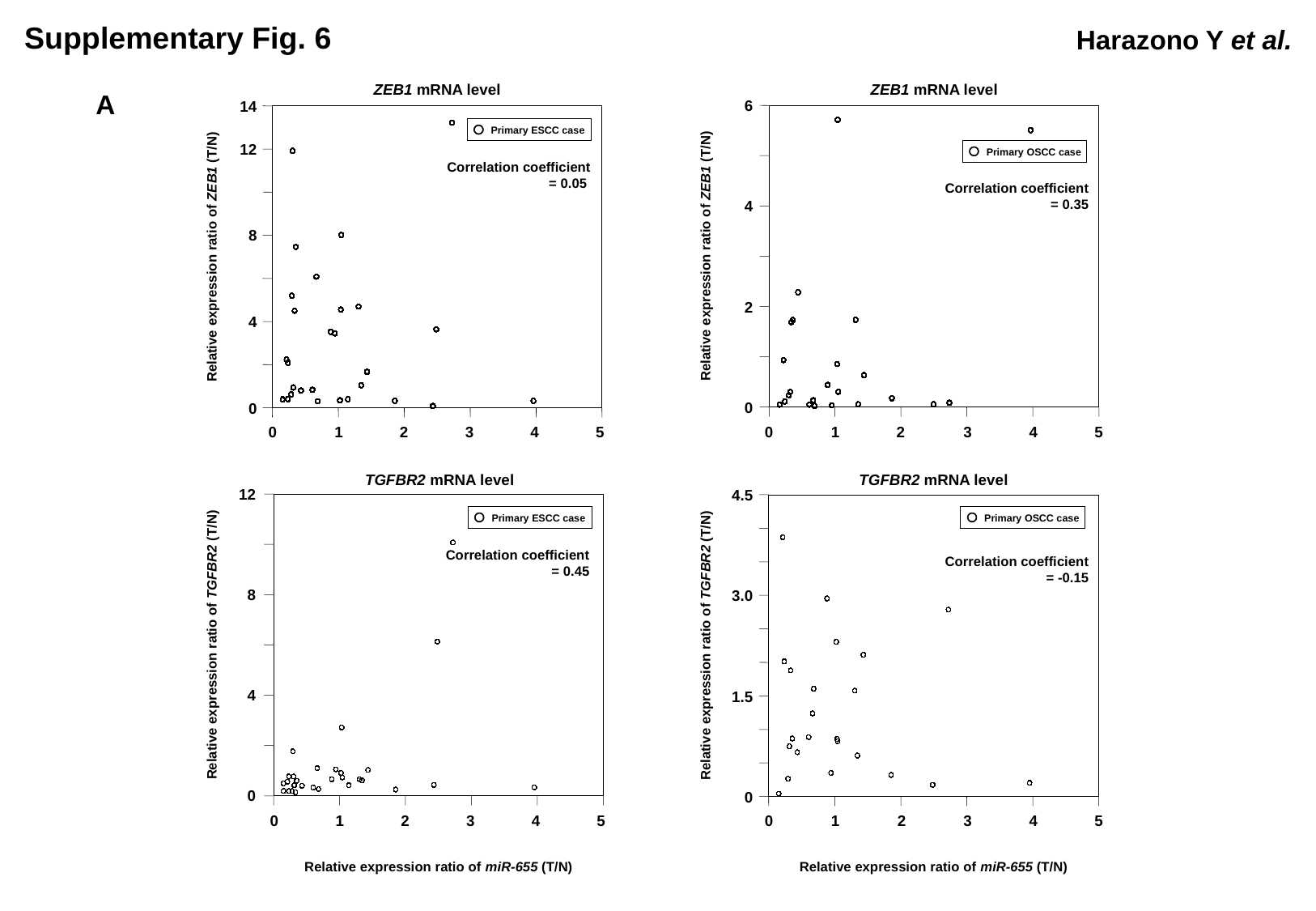

Supplementary Fig. 6
Harazono Y et al.
ZEB1 mRNA level
ZEB1 mRNA level
A
6
14
Primary ESCC case
12
Primary OSCC case
Correlation coefficient
 = 0.05
Correlation coefficient
 = 0.35
4
8
Relative expression ratio of ZEB1 (T/N)
Relative expression ratio of ZEB1 (T/N)
2
4
0
0
0
1
2
3
4
5
0
1
2
3
4
5
TGFBR2 mRNA level
TGFBR2 mRNA level
12
4.5
Primary ESCC case
Primary OSCC case
Correlation coefficient
 = 0.45
Correlation coefficient
 = -0.15
8
3.0
Relative expression ratio of TGFBR2 (T/N)
Relative expression ratio of TGFBR2 (T/N)
4
1.5
0
0
0
1
2
3
4
5
0
1
2
3
4
5
Relative expression ratio of miR-655 (T/N)
Relative expression ratio of miR-655 (T/N)

## Slide 2
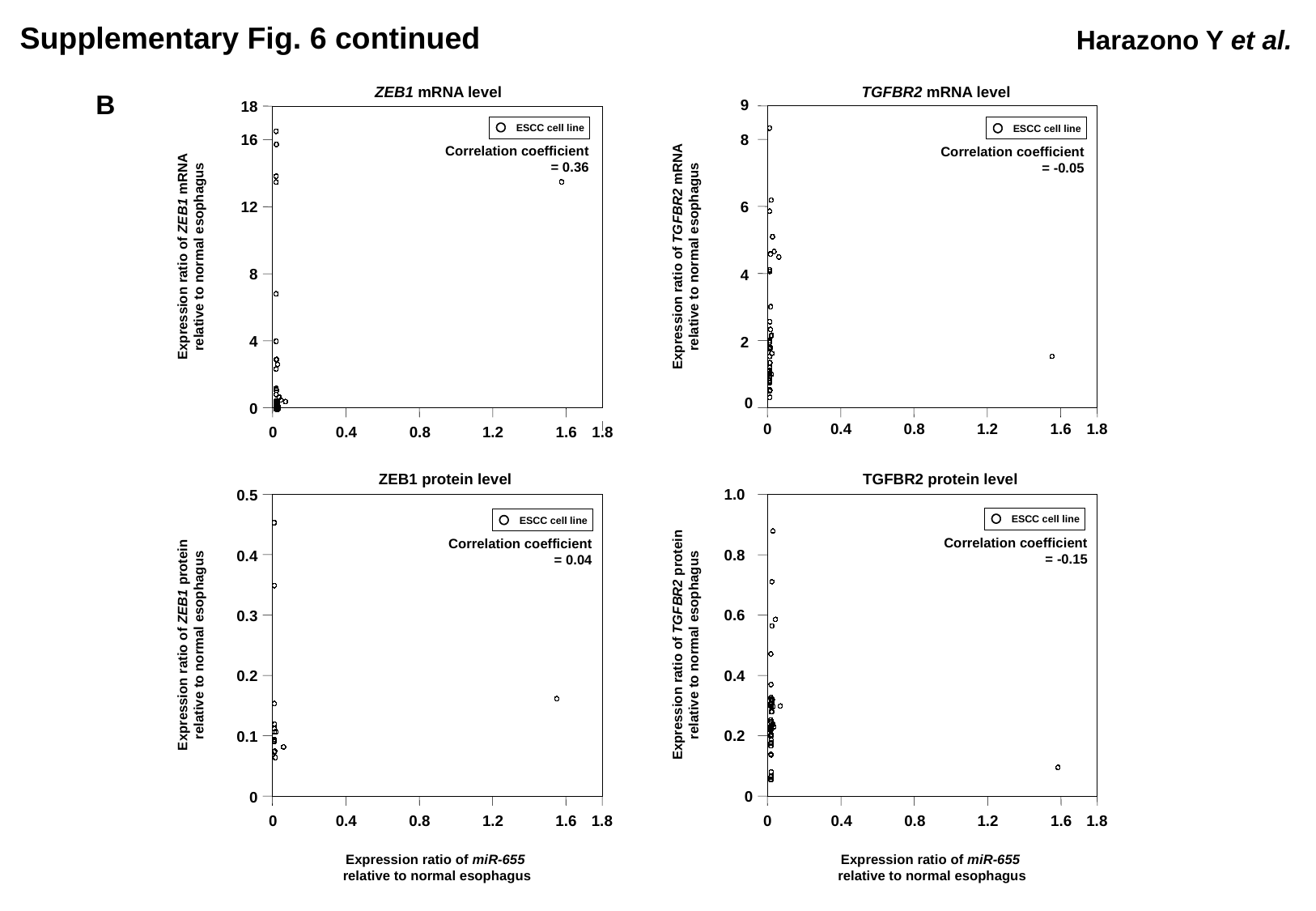

Supplementary Fig. 6 continued
Harazono Y et al.
ZEB1 mRNA level
TGFBR2 mRNA level
B
9
18
ESCC cell line
ESCC cell line
16
8
Correlation coefficient
 = 0.36
Correlation coefficient
 = -0.05
6
12
Expression ratio of TGFBR2 mRNA
relative to normal esophagus
Expression ratio of ZEB1 mRNA
relative to normal esophagus
8
4
4
2
0
0
0
0.4
0.8
1.2
1.6
1.8
0
0.4
0.8
1.2
1.6
1.8
ZEB1 protein level
TGFBR2 protein level
1.0
0.5
ESCC cell line
ESCC cell line
Correlation coefficient
 = -0.15
Correlation coefficient
 = 0.04
0.8
0.4
0.6
0.3
Expression ratio of ZEB1 protein
relative to normal esophagus
Expression ratio of TGFBR2 protein
relative to normal esophagus
0.4
0.2
0.2
0.1
0
0
0
0.4
0.8
1.2
1.6
1.8
0
0.4
0.8
1.2
1.6
1.8
Expression ratio of miR-655
relative to normal esophagus
Expression ratio of miR-655
relative to normal esophagus
