# Supplementary material for: miR-655 Is an EMT-Suppressive MicroRNA Targeting ZEB1 and TGFBR2
Source: PLoS One. 2013 May 14;8(5):e62757. doi: 10.1371/journal.pone.0062757 (PMC3653886; doi:10.1371/journal.pone.0062757)
Supplement: Figure S7 — TaqMan real-time RT-PCR analysis (Upper) and Western blot (Lower) analysis for ZEB1 (left) and TGFBR2 (right) in Panc1, KP1N and MDA-MB-231 cells 96 hours after transfection of 10 nM of ds-NC or ds-miR-655 (Thermo Scientific Dharmacon). (PPT) [file pone.0062757.s007.ppt]

## Slide 1
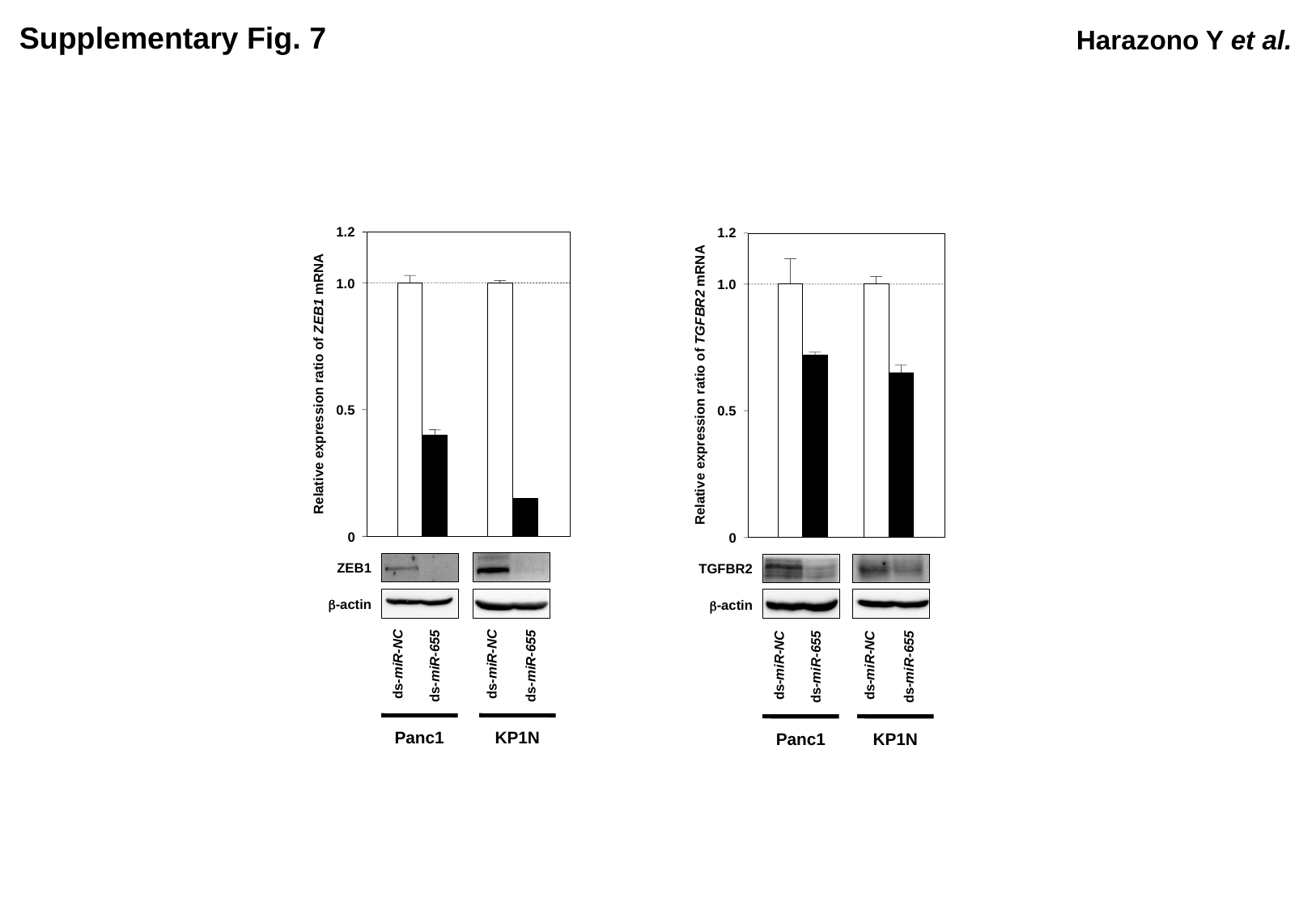

Supplementary Fig. 7
Harazono Y et al.
1.2
1.2
1.0
1.0
Relative expression ratio of ZEB1 mRNA
Relative expression ratio of TGFBR2 mRNA
0.5
0.5
0
0
ZEB1
TGFBR2
β-actin
β-actin
ds-miR-NC
ds-miR-NC
ds-miR-NC
ds-miR-NC
ds-miR-655
ds-miR-655
ds-miR-655
ds-miR-655
Panc1
KP1N
Panc1
KP1N
