# Supplementary material for: miR-655 Is an EMT-Suppressive MicroRNA Targeting ZEB1 and TGFBR2
Source: PLoS One. 2013 May 14;8(5):e62757. doi: 10.1371/journal.pone.0062757 (PMC3653886; doi:10.1371/journal.pone.0062757)
Supplement: Figure S8 — A, Complementary miR-655 seed sequence and PCR region in the 3′UTR of ZEB1 (Upper) and TGFBR2 (Lower). These sites were analyzed using microRNA.org and Target Scan Human 6.2. B, Results of luciferase reporter assays in Panc1 cells 48 hours after cotransfection of pMIR-REPORT luciferase vectors containing wild-type of ZEB1 or TGFBR2 for miR-655, ds-miR-655 or ds-NC, and the pRL-CMV internal control vector. Asterisks (*), statistical analysis with the Mann-Whitney U test. (PPT) [file pone.0062757.s008.ppt]

## Slide 1
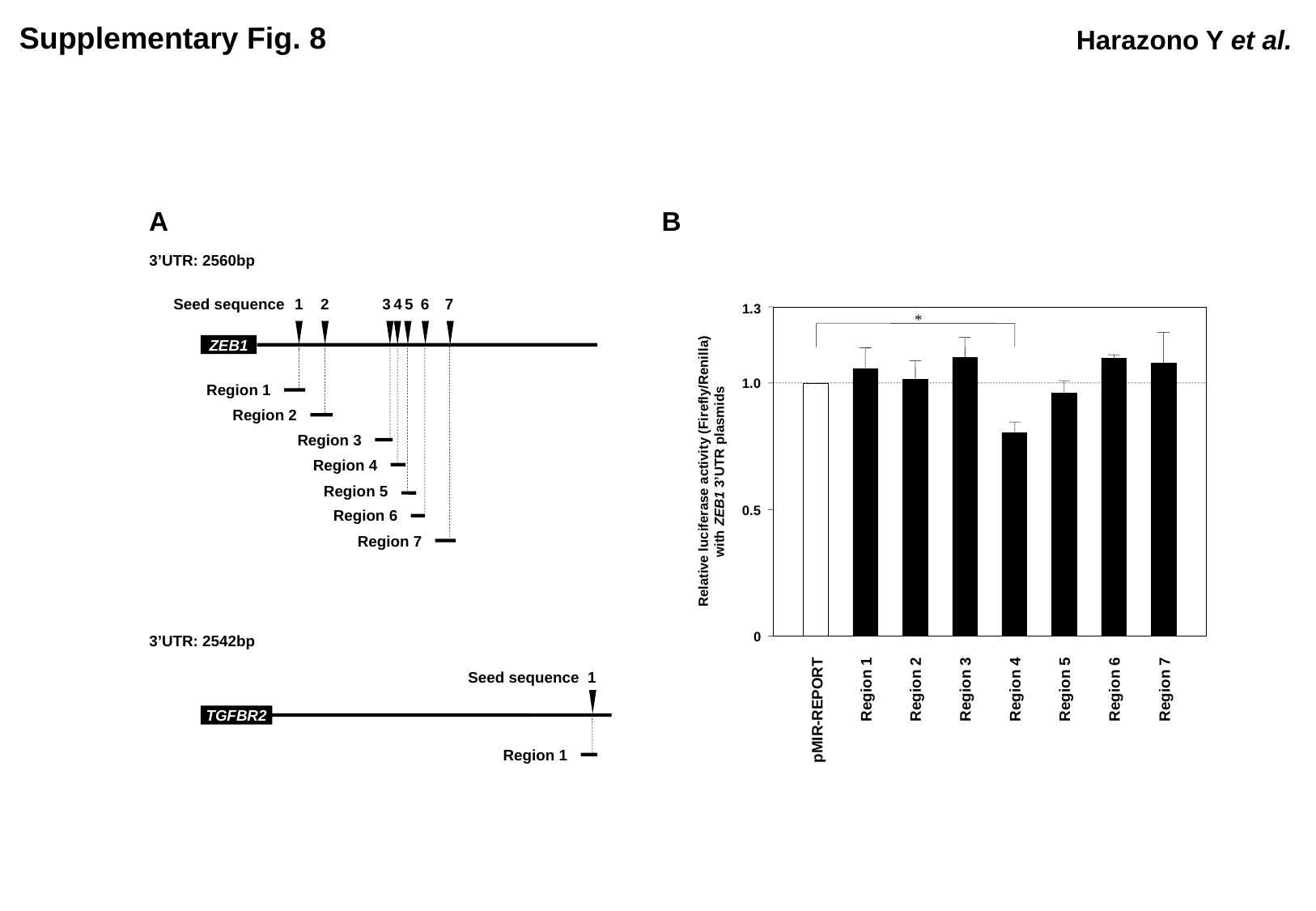

Supplementary Fig. 8
Harazono Y et al.
A
B
3’UTR: 2560bp
Seed sequence
1
2
3
4
5
6
7
1.3
*
ZEB1
1.0
Region 1
Region 2
Region 3
Region 4
Relative luciferase activity (Firefly/Renilla)
with ZEB1 3’UTR plasmids
Region 5
0.5
Region 6
Region 7
0
3’UTR: 2542bp
Seed sequence
1
Region 1
Region 2
Region 3
Region 4
Region 5
Region 6
Region 7
pMIR-REPORT
TGFBR2
Region 1
