# Supplementary material for: miR-655 Is an EMT-Suppressive MicroRNA Targeting ZEB1 and TGFBR2
Source: PLoS One. 2013 May 14;8(5):e62757. doi: 10.1371/journal.pone.0062757 (PMC3653886; doi:10.1371/journal.pone.0062757)
Supplement: Figure S9 — TaqMan real-time RT-PCR analysis for CDH1/E-cadherin (left) and PAI-1 (right) in KP1N cells 96 hours after transfection of 10 nM of ds-NC or ds-miR-655 (Ambion). Cells were analyzed 72 hours after treatment with or without TGF-b (5 ng/ml) and transfection with ds-miR-655 or ds-NC, simultaneously. (PPT) [file pone.0062757.s009.ppt]

## Slide 1
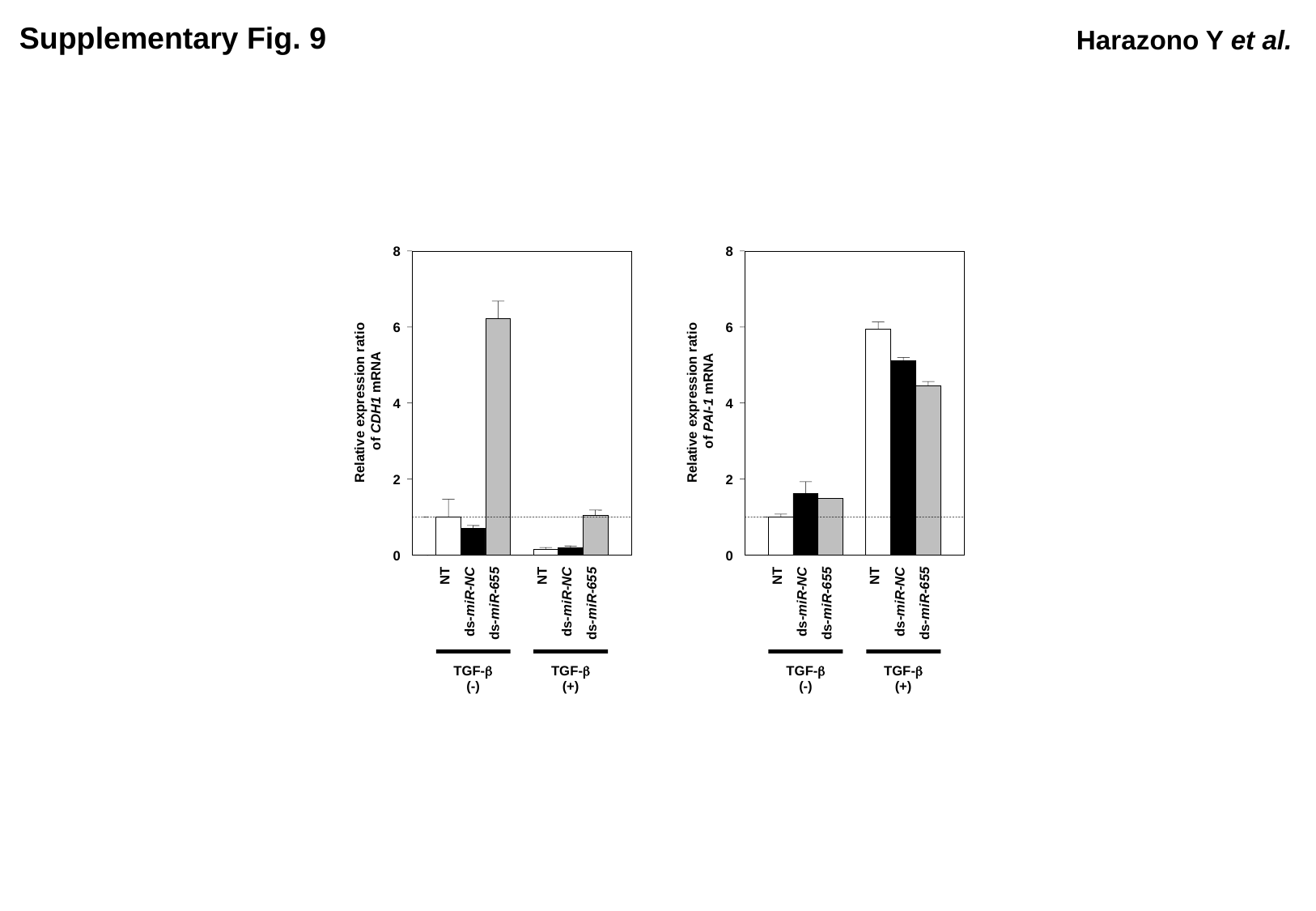

Supplementary Fig. 9
Harazono Y et al.
8
8
6
6
Relative expression ratio
 of CDH1 mRNA
Relative expression ratio
 of PAI-1 mRNA
4
4
2
2
0
0
NT
NT
NT
NT
ds-miR-NC
ds-miR-NC
ds-miR-NC
ds-miR-NC
ds-miR-655
ds-miR-655
ds-miR-655
ds-miR-655
TGF-β
(-)
TGF-β
(+)
TGF-β
(-)
TGF-β
(+)
